# Desymmetrization of Malonic Monoesters and Malonic Acids via Enantioselective Catalytic C(sp^3^)─H Oxidation

**DOI:** 10.1002/anie.1030779

**Published:** 2026-02-17

**Authors:** Nikos Siakavaras, Arnau Call, Massimo Bietti, Miquel Costas

**Affiliations:** ^1^ Institut De Química Computacional i Catàlisi (IQCC) and Departament de Química Universitat De Girona, Campus Montilivi Catalonia Spain; ^2^ Dipartimento Di Scienze e Tecnologie Chimiche Università “Tor Vergata” Via Della Ricerca Rome Italy

## Abstract

Malonate derivatives are readily available starting materials widely employed in the synthesis of bioactive compounds. Herein, we report a novel catalytic protocol for the direct desymmetrization of malonic acids via enantioselective C(sp^3^)─H bond functionalization. Highly enantioselective (up to >99% ee) *γ*‐ and *δ*‐ C─H bond lactonization of readily available malonic acid monoesters and malonic acids is achieved, using manganese catalysts and hydrogen peroxide as the oxidant. Owing to the ease and versatility of malonic acid derivative synthesis, combined with the potential post‐oxidation elaboration, this methodology overcomes the inherent lack of reactivity of the electron‐poor C(sp^3^)─H bonds of this class of substrates against electrophilic oxidants to provide straightforward access to a broad range of quaternary stereocenters that can be orthogonally manipulated.

## Graphical abstract

Manganese‐catalyzed enantioselective C(sp^3^)─H lactonization with hydrogen peroxide enables the direct desymmetrization of malonic monoesters and malonic acids, providing highly enantioenriched γ‐ and δ‐lactones (up to >99% ee)

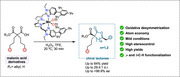

## 1 Introduction

**FIGURE 1:**
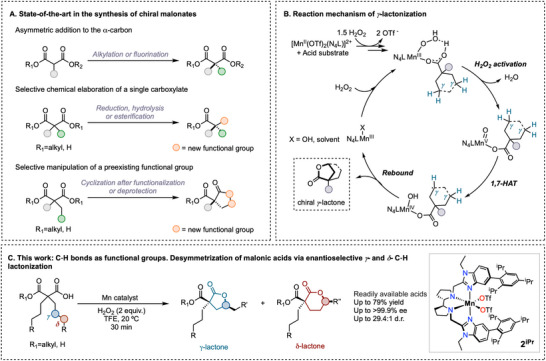
(A) Reported strategies for malonate desymmetrization. (B) General mechanism for *γ*‐C−H bond lactonization catalyzed by manganese complexes. N_4_L stands for a tetradentate ligand. (C) Summary of the main features of this work.

The inherent relationship between molecular shape and activity has driven the development of new synthetic methodologies for chiral molecules, particularly those containing densely functionalized quaternary stereocenters [1]. Desymmetrization reactions can be used as a powerful synthetic tool for accessing enantioenriched scaffolds using cheap, simple and easily available starting materials that can be further elaborated for the synthesis of biologically active compounds [2]. While desymmetrization reactions have proven effective for a wide range of substrates, the synthetic utility of these methods is ultimately dictated by the availability of prochiral precursors and the chemical versatility of the resulting chiral products [3]. Within this framework, malonates and their derivatives rank among the best candidates as they are highly accessible starting materials, allow for facile orthogonal chemical manipulation [4], and have featured prominently in numerous retrosynthetic pathways [5, 6, 7, 8]. Given their broad applicability, various synthetic strategies have been developed and used widely for single step transformation to chiral structures (Figure 1A), including: (i) asymmetric *α*‐addition of *α*‐monosubstituted malonate diesters [9, 10, 11], (ii) selective chemical manipulation of one of the two carboxylate functionalities in prochiral *α*,*α*‐disubstituted malonates; [12, 13, 14, 15, 16, 17, 18, 19, 20], and (iii) selective elaboration of a single ester group through cyclization strategies [21, 22, 23, 24]. While these desymmetrization procedures are well established, they are essentially limited in their ability to differentiate preexisting functional groups to generate a stereogenic center at the *α*‐position. Notably, the significant challenge of accessing high‐value chiral malonic derivatives via enantioselective C─H functionalization remains unexplored.

Building on the potential of asymmetric *γ*‐C─H bond lactonization reactions catalyzed by chiral tetradentate manganese complexes in the presence of H_2_O_2_ for desymmetrizing simple carboxylic acids [25, 26, 27, 28], we envisioned that such a directed oxidation strategy could be a practical solution to this challenge. The proposed mechanism involves biomimetic heterolytic cleavage of H_2_O_2_ to generate manganese‐oxo‐carboxylato species [29, 30, 31, 32], which can oxygenate nonactivated C(*sp^3^*)─H bonds via an enantiodiscriminative 1,7‐HAT/rebound mechanism (Figure 1B). As these reactions proceed through coordination of the carboxylic acid substrate to the Mn‐center, this approach may provide a strategic means of desymmetrizing the two *α,α−*dialkyl chains in malonic acid monoesters and malonic acids, thereby enabling direct access to densely functionalized, orthogonally versatile chiral oxygenated lactones. However, this approach presents inherent challenges. Malonic acid derivatives are electronically deactivated systems, a property that can be further accentuated in fluorinated alcohols [33, 34], rendering their C(sp^3^)─H bonds notoriously difficult to break by radical and radical‐like electrophilic oxidants. Moreover, their strong chelating ability toward transition‐metal centers may promote catalyst inhibition or undesired off‐cycle pathways.

Herein, we describe the successful development of a strategy for the enantioselective catalytic desymmetrization of *α,α−*dialkyl malonic acid monoesters and malonic acids via a highly enantioselective (up to >99.9% ee) directed C─H oxidation using manganese catalysts and H_2_O_2_ as the oxidant (Figure 1C). This protocol produces *γ−* and *δ−*lactones in good yields, with tunable site‐selectivity leveraging the structural modularity of the substrates. Moreover, by employing *α,α−*dialkyl malonic acids, the system enables direct access to chiral spirocyclic bilactones through two sequential *γ−*lactonization reactions. The utility of these products is further demonstrated through chemical elaboration of the malonate group, while preserving the stereochemical information established in the lactonization step.

## 2 Results and Discussion

**FIGURE 2:**
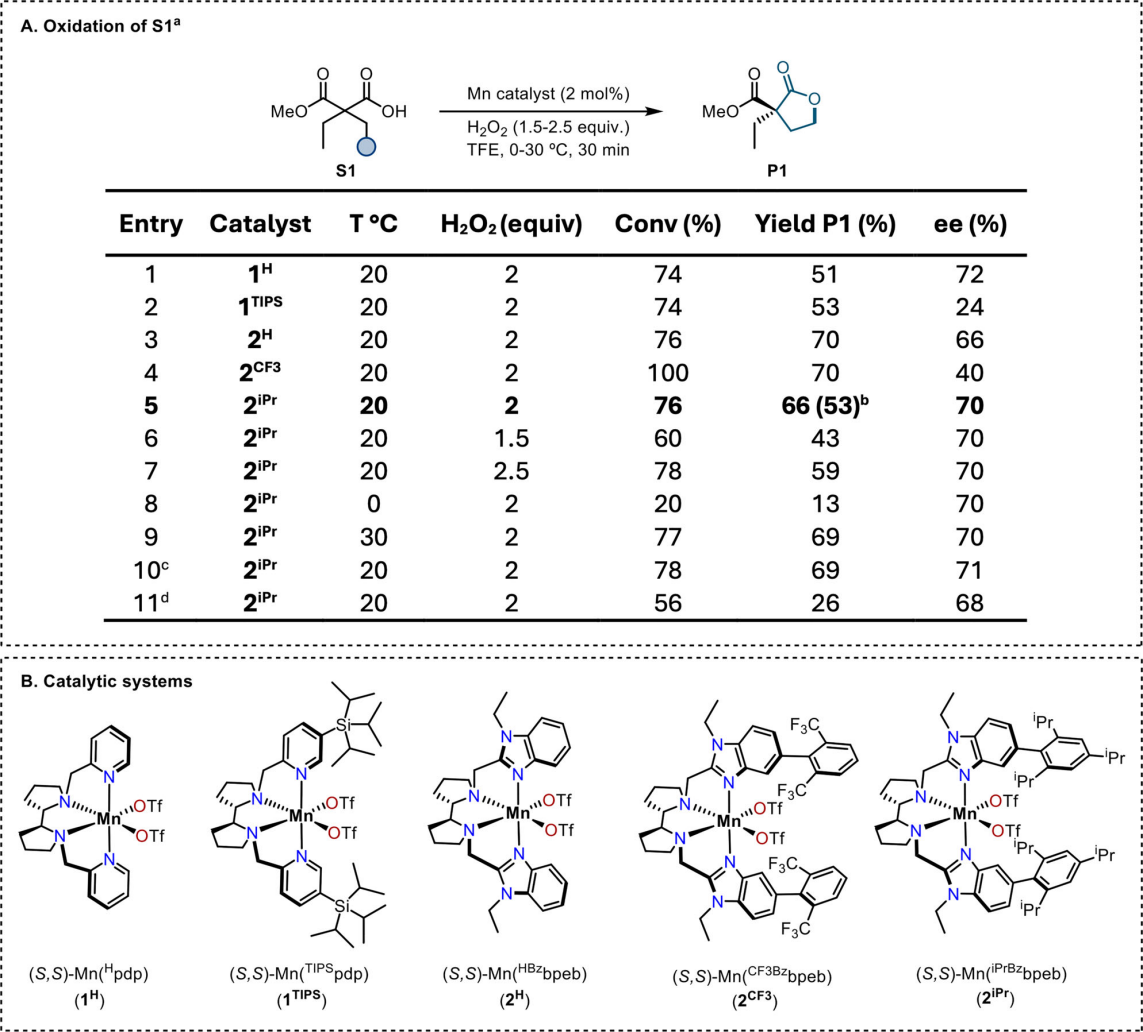
Initial experiments and optimization for the enantioselective oxidative desymmetrization of **S1**. ^a^Reaction conditions: substrate (25 mM) and Mn catalysts (2 mol%) were initially dissolved in TFE, and 2 equiv. of H_2_O_2_ (0.45 M solution in TFE) were independently delivered over 30 min with a syringe pump, at 20°C, unless otherwise indicated. After the addition, the reaction mixture was left under magnetic stirring for additional 30 min. Workup is indicated in the Supporting Information. Conversion, yield, and ee were determined by GC analysis of two or three different runs with biphenyl as internal standard. ^b^ Yield of the isolated product. ^c^Using HFIP as the solvent. ^d^ Using MeCN as the solvent.

Building on our recent reported procedures for *γ−*lactonization [28, 32, 35], 2‐ethyl‐2‐(methoxycarbonyl)butanoic acid (**S1**) bearing two enantiotopic *γ−*methyl groups was selected as a model substrate to initiate the exploration of enantioselective desymmetrization of malonic acid monoesters (Figure 2). This compound was selected because of the inherent challenge of oxidizing a primary C─H bond via HAT, due to high BDE for this class of C─H bonds and electronic deactivation exerted by the two carboxylate moieties. Slow addition of 2 molar equivalents of H_2_O_2_ (added via syringe pump over 30 min) as the oxidant to a 2,2,2‐trifluoroethanol (TFE) solution of **S1** and (*S,S*)‐Mn(^H^PDP) (**1^H^**) catalyst (2 mol%) at 20 °C resulted in 74% substrate conversion, yielding lactone **P1**, resulting from the selective C(*sp^3^*)‐H oxidation of one of the two methyl groups, in 51% yield with a notable enantioselection (72% ee) (entry 1). No additional oxidation product was detected by gas chromatography analysis. Notably, the enantioselection observed for **P1** arises from the discrimination of the two enantiotopic primary *γ−*carbons, which leads to the generation of an *α*‐quaternary stereocenter. Intriguingly, the use of a bulkier catalyst (*S,S*)‐Mn(^TIPS^PDP) (**1^TIPS^**) afforded **P1** in comparable yield, but the enantioselectivity significantly dropped to 24% ee (entry 2).

Changing the nature of the ligand and moving to benzimidazole‐based manganese catalysts, the simple (*S,S*)‐Mn(bpeb) catalyst (**2^H^**) increased **P1** yield (70%), while a slight decrease in enantioselection was observed (66% ee, entry 3). The addition of aryl groups at position 5 of the benzimidazole rings has been proposed as a strategy for structural shaping of highly reactive chiral Mn‐oxo species, which improves both yield and enantioselectivity [28, 36]. Following this strategy, the sterically encumbered catalysts (*S,S*)‐Mn(^CF3^bpeb) (**2^CF3^**) and (*S,S*)‐Mn(^iPr^bpeb) (**2^iPr^**) were tested in the model reaction. This led to the identification of **2^iPr^** as the most effective catalyst, restoring the ee to 70%, with a moderate 66% yield (53% yield of the isolated product, entry 5). As there is no significant difference in enantioselectivity between catalysts **1^H^** and **2^iPr^**, we selected **2^iPr^** as the optimal catalyst due to the higher product yield. Moreover, **2^iPr^** was found to be more general across other substrates (vide infra) and was used for further optimization. Reducing the amount of H_2_O_2_ to 1.5 equivalents decreased **P1** yield to 43% (entry 6), whereas increasing it to 2.5 equivalents showed no significant improvement (59% yield, entry 7). The reaction proved more sensitive to temperature; lowering it to 0°C drastically reduced the yield to 13% (entry 8), while increasing it to 30°C maintained a comparable yield of 69%, without affecting enantioselectivity (70% ee, entry 9). This decrease in reaction efficiency at lower temperature is in line with the results obtained for the *γ*‐lactonization at deactivated primary positions catalyzed by the same Mn‐complexes [32]. Solvent screening showed that replacing TFE with 1,1,1,3,3,3‐hexafluoro‐2‐propanol (HFIP) had no impact on yield and enantioselectivity (entry 10). Given its lower cost, TFE was therefore considered the most appropriate solvent. In contrast, switching to acetonitrile (MeCN) resulted in a notable drop in yield (26%) and slightly reduced enantioselectivity (68% ee, entry 11), indicating that fluorinated alcohols are the most effective solvents for this desymmetrization, likely due to their ability to enhance the reactivity of manganese‐oxo species, as previously reported [27, 32, 37].

**FIGURE 3:**
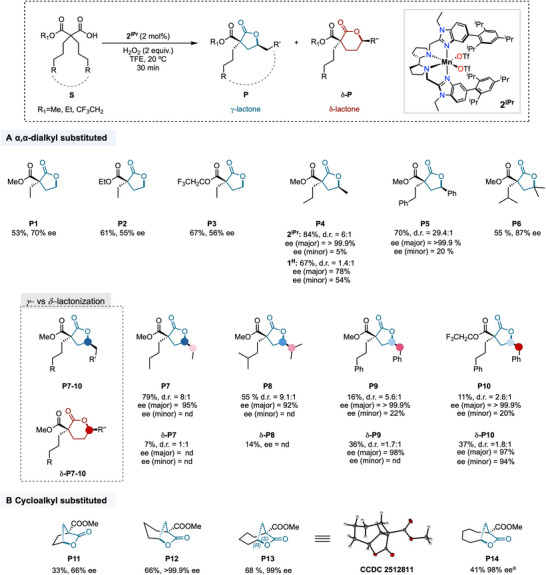
Substrate scope using *α,α*‐disubstituted malonic acid monoesters. (A) Dialkyl substituents. (B) Cycloalkyl substituents. Reaction conditions: substrate (25 mM) and **2^iPr^** (2 mol %) were initially dissolved in TFE, and 2 equiv. of H_2_O_2_ (0.45 M solution in TFE) were independently delivered over 30 min with a syringe pump, at 20°C. After addition, the reaction mixture was left under magnetic stirring for an additional 30 min. ^a^ Yield of **P11** is diminished due to the formation of *γ*‐hydroxyacid and *γ*‐ketoacid (∼10% total yield) without cyclization to lactone product due to the five‐member ring unfavored conformation. ^b^ Yield of **P14** is diminished due to the formation of unidentified overoxidation byproducts. Yields are based on the amount of isolated oxidation product obtained from larger‐scale reactions (see the supporting information) d.r. = diastereomeric ratio. ee = enantiomeric excess. nd = not determined.

The reaction enantioselectivity proved highly sensitive to the nature of the ester group. For example, replacing the methyl ester with either an ethyl (**S2**) or a trifluoroethyl one (**S3**) led to a marked decrease in enantioinduction, affording the corresponding lactones **P2** and **P3** with 55% and 56% ee, respectively, although with a slight increase in yield (61% and 67%). Methyl monoester was therefore selected as the standard structural unit for further desymmetrization studies on malonic acid monoesters, both because of its ease of manipulation and its good overall performance. With the optimal conditions in hand, we then evaluated the oxidative desymmetrization of a series of different *α,α−*dialkyl substituted malonic acid monoesters (the overall reaction and the corresponding products are summarized in Figure 3A).

Extending the alkyl chains by one carbon, as in **S4,** markedly improved reaction efficiency, affording *γ−*lactone **P4** in 84% isolated yield. This enhancement can be attributed to the intrinsically lower bond dissociation energy (BDE) of the targeted *γ−*methylenic C─H bonds compared to the primary ones displayed by **S1**‐**S3**. In addition, the reaction shows good diastereocontrol (d.r. = 6:1) and outstanding enantiocontrol (>99.9% ee for the major diastereomer), with the diastereoselectivity arising from the discrimination of the two C─H bonds on the same *γ−*carbon. In comparison, desymmetrization of **S4** using **1^H^** in place of **2^iPr^** deteriorates catalytic activity in all aspects (67% yield, d.r. = 1.4, 78% ee for the major enantiomer), highlighting **2^iPr^** as the preferred catalyst for highly enantioselective *γ−*lactonization at secondary sites [28]. Desymmetrization of malonic acid monoester **S5** bearing weaker benzylic *γ*‐C─H bonds leads to the exclusive formation of *γ*‐lactone **P5** in good yield (70%). Remarkably, the system shows high compatibility with substrates bearing aromatic groups, which, under these oxidative conditions, are typically prone to undesired aromatic oxidation leading to catalyst inhibition [35]. Notably the phenyl group plays a crucial role in maximizing diastereocontrol, delivering an outstanding 29.4:1 d.r., with the major diastereoisomer obtained again in enantiopure form (>99.9% ee). Desymmetrization of the malonic acid monoester **S6** bearing tertiary *γ*‐C─H bonds led to the desired tertiary *γ*‐lactone (**P6**) in moderate yield (55%) but relatively high enantioselectivity (87% ee). The result is significant because it means that the present methodology enables direct access to chiral *γ*‐lactones by enantioselective tertiary C─H bond functionalization, a pioneering transformation that remains inaccessible with classical palladium‐catalyzed C─H activation strategies where steric hindrance prevents formation of the required organometallic intermediates [38, 39, 40, 41].

Further elongation of the alkyl chains by an additional methylenic unit (**S7**) results in no erosion of the diastereomeric ratio (d.r. = 8), while both the *γ*‐lactone yield (**P7**, 79%) and enantioselectivity for the major diastereomer (95% ee) remained at very satisfactory levels. Unexpectedly, analysis of the crude reaction mixture revealed the formation of a six‐membered δ‐lactone product (**δ‐P7**) in 7% yield, arising from *δ*‐C─H bond lactonization with no diastereocontrol (d.r. = 1). Although the *γ*‐methylenic site is expected to be electronically more deactivated than the *δ*‐ one, the dominant oxidation of the former site (**P7**/** δ‐P7**∼11) can be mainly attributed to kinetic factors [32].

Notwithstanding, when the desymmetrization occurs in substrates bearing weaker tertiary *δ*‐C─H bonds (**S8**), *γ*‐lactone **P8** was formed in 55% yield, but a more significant amount of *δ*‐lactone **δ‐P8** was also observed (14% yield, **P8**/**δ‐P8 **= 3.9). Following the established trend of increasing d.r. with increasing alkyl chain complexity (**P7**>**P4**), a further increase in diastereocontrol is observed for **P8** (d.r. = 9.1), along with excellent enantioselectivity for the major diastereomer (92% ee). In the presence of a benzylic *δ*‐methylenic site as in **S9**, *δ*‐lactone prevails over the *γ*‐ one (**P9**/** δ‐P9** = 0.44). Formation of **P9** exhibited a good diastereomeric ratio (5.6:1) with outstanding levels of enantioselectivity (>99.9% ee) for the major diastereomer, while the minor diastereomer suffers from low enantiocontrol (22% ee) as observed in **P4** (5% ee) and **P5** (20% ee). Although the oxidation of the *δ*‐position (**δ‐P9**) occurs with modest diastereocontrol (d.r. 1.7), the major diastereoisomer is also obtained in enantiopure form (>99.9% ee), and based on previous computational studies [32] which indicated comparable energy barriers for carboxylic acid directed 1,7 and 1,8‐HAT pathways, it is reasonable to suggest that the oxidation at the *δ*‐position occurs through the later pathway. Asymmetric *δ*‐C─H bond functionalization reactions remain limited in literature, and are mostly restricted to nitrogen transfer [42, 43, 44, 45] and arylation [46, 47] reactions. To the best of our knowledge, the present system represents the first example of a highly enantioselective *δ*‐C─H bond oxygenation. Although **2^iPr^** was previously reported to exhibit exquisite selectivity for the oxidation at *γ*‐positions in carboxylic acids, even in the presence of a priori more reactive *δ*‐ and *β*‐C─H bonds [28], the change in site‐selectivity observed in malonic acid monoesters benefits from the contribution of a stronger electronic deactivation of the *γ*‐C─H bonds. This intrinsic electronic effect is enforced by polarity enhancement induced by solvent hydrogen bonding to the electron‐withdrawing malonate group [34, 37, 48]. Consequently, the *γ/δ*‐lactone ratio correlates with the relative decrease in *δ*‐C─H bond BDE **S4** (1^ο^)>**S7** (2°)>**S8** (3°)>**S9** (benzylic) [49, 50]

Building on the unique opportunity offered by malonate derivatives to modulate site‐selectivity through polarity enhancement, we sought to further enhance the preference for *δ*‐lactone formation by increasing electronic deactivation. Crucially, replacing the simple methyl ester with a more electron‐withdrawing trifluoroethyl one (**S10**) maximized selectivity for benzylic *δ*‐C─H bond oxidation (**P10**/** δ‐P10 **= 0.3), albeit with some erosion in yield and diastereoselectivity for the *γ*‐lactones. However, the major *γ*‐lactone diastereomer retained enantiopurity (**P10**, >99.9% ee). The modest diastereoselectivity observed for **δ‐P9** and **δ‐P10** formation (d.r. = 1.7‐1.8) underscores the inherent challenges in achieving high diastereoselectivity in *δ*‐lactonization reactions. Nevertheless, these results reinforce the effectiveness of directed oxidation strategies, as reflected in the exceptionally high enantiodiscrimination values observed for both **δ‐P10** diastereomers (97% for the major and 94% for the minor). We note on passing that attaining high enantioselectivities in such strongly electronically deactivated systems is particularly challenging, owing to the sensitivity of the enantiodetermining HAT step to the electronics of targeted C(*sp^3^*)─H bonds, as recently supported by statistical modeling analysis [51, 52]

Having established the protocol for the desymmetrization of acyclic *α*,*α*‐dialkyl substituted malonic acid monoesters, we next sought to expand its scope toward the synthesis of fused bicyclic lactones from *α,α−*cycloalkyl malonic acid monoesters (**S11**‐**S14**) (Figure 3B). The oxidation of the five‐membered ring substrate **S11** proceeded site‐selectively, affording *γ*‐lactone **P11** in 33% yield as a single diastereoisomer, with moderate enantioselectivity (66% ee). The formation of the corresponding *γ*‐hydroxyacid and *γ*‐ketoacid (10% combined yield), arising from oxidation at the *γ*‐C─H bonds, is attributed to conformational constraints imposed by the cyclopentane ring [35]. In contrast, enantiocontrol was fully restored (>99.9% ee) when the oxidation was performed on the six‐membered ring derivative **S12**, affording the desired bicyclic *γ*‐lactone **P12**, a structural motif commonly found in steroid scaffolds [53, 54], in a valuable 66% yield. Furthermore, expansion of the flanking ring to 7 and 8 carbons furnished the bicyclic *γ*‐lactones **P13**‐**P14**, hybrids between 8‐ and 9‐membered *ζ*‐enantholactone and *η*‐octalactone with 5‐membered *γ*‐butyrolactone, respectively, all obtained with exquisite levels of site‐ and enantiocontrol. It is noteworthy that the stereoselectivity of **P13**‐**P14** arises from the selective oxidation of a single C─H bond among twelve or fourteen methylenic C−H bonds, respectively.

**FIGURE 4:**
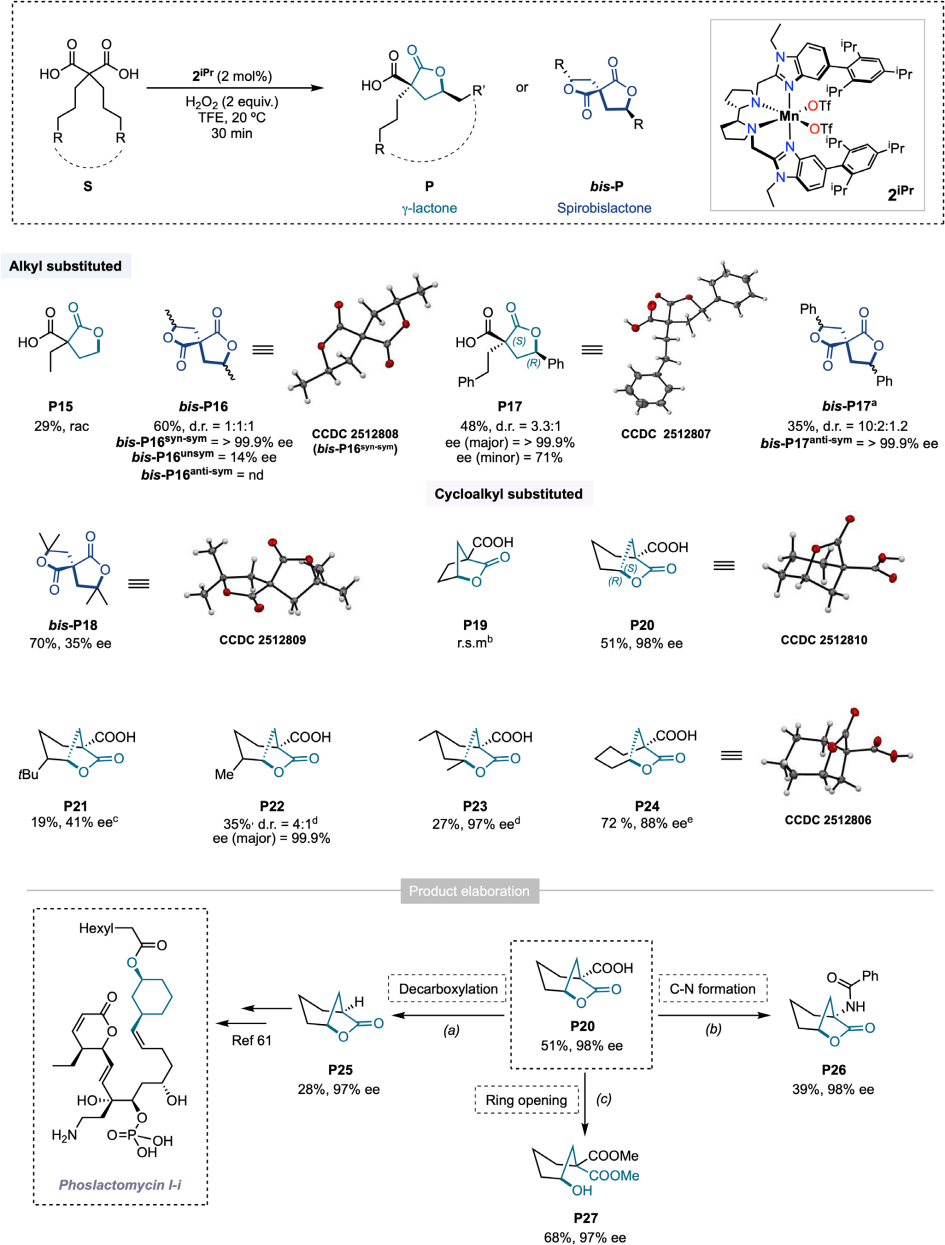
Substrate scope using *α,α*‐disubstituted malonic acids. Reaction conditions: substrate (25 mM) and **2^iPr^** (2 mol %) were initially dissolved in TFE, and 2 equiv. of H_2_O_2_ (0.45 M solution in TFE) were independently delivered over 30 min with a syringe pump, at 20°C. After the addition, the reaction mixture was left under magnetic stirring for an additional 30 min. ^a^The crude solution was evaporated, filtered through a silica plug using ethyl acetate as eluent. After vacuum removal of solvent until complete dryness, the crude solid was again subjected to reaction conditions. ^b^Recovery of starting material. ^c^ **2^H^** (2 mol%) was used as a catalyst. ^d^ After the first addition, a second portion of H_2_O_2_ (2 equiv, over 30 min) and **2^iPr^** (2 mol%) were added. ^e^ A few drops of MeCN were added until complete substrate solubilization. Yields are based on the amount of isolated oxidation product obtained from larger‐scale reactions (see the supporting information) d.r. = diastereomeric ratio. ee = enantiomeric excess. Product elaboration: (a) *p*‐TsOH acid monohydrate (2 equiv.), DMSO, 120°C, 3 days. (b) (i) SOCl_2_, 2 h, r.t., (ii) NaN_3_, toluene, 2 h, r.t., (iii) THF, 1 h, reflux, (iv) benzoyl chloride (2 equiv.), K_2_CO_3_ (2 equiv.), H_2_O, overnight. (C) MeOH, in the presence of a few drops of concentrated H_2_SO_4_, 70°C, overnight.

The generality of the methodology for the desymmetrization of malonic acid monoesters was next examined, extending the reaction to a series of *α,α−*disubstituted malonic acids. C─H bond lactonization of dicarboxylic acids has previously been reported only by Yu and coworkers, to afford racemic lactone acids via Pd catalysis [55]. The formation of chiral *γ*‐lactones from diacids has otherwise only been achieved through asymmetric hydrogenation processes catalyzed by Rh or Ir complexes [56]. Oxidation of diacid **S15**, an analogue of **S1**, furnished the corresponding lactone acid (**P15**) in 29% yield without enantiodiscrimination (Figure 4). The double lactonization product was not detected even after two consecutive oxidation attempts, reflecting the difficulty of promoting a second C─H bond oxidation on the strongly electronically deactivated oxidized product **P15**. Nevertheless, oxidation of diacid **S16**, which contains two *γ*‐methylenic positions, afforded three bicyclic spirolactones (***bis‐*P16**) in a statistical 1:1:1 diastereoisomeric ratio, in an overall 60% yield (See SI for details). Importantly, the one‐pot sequential double lactonization process produced one diastereoisomer with an exceptional enantiomeric excess of >99.9%, whereas the second diastereoisomer exhibited much lower enantiodiscrimination (14% ee). Nonetheless, the high levels of enantioselectivity observed for one diastereoisomer demonstrate the feasibility of stereo‐controlled desymmetrization of diacid scaffolds, offering a straightforward strategy for constructing chiral spirocyclic architectures bearing quaternary stereocenters, common in natural product frameworks [57]. Desymmetrization of dicarboxylic acid **S17** bearing *γ*‐benzylic sites, predominantly afforded the monolactone product (**P17**), with only trace amounts of the corresponding bilactone ***bis‐*P17** detected. Under standard reaction conditions, **P17** was isolated in 48% yield with a diastereomeric ratio of 3.3:1. The major diastereoisomer was obtained in essentially enantiopure form, whereas the minor diastereoisomer exhibited a noteworthy enantiomeric excess (71% ee). Notably, when the crude mixture was passed through a silica plug and subsequently subjected to the reaction conditions, **P17** underwent conversion to the corresponding ***bis‐*P17** product in a d.r = 10:2:1.2, and overall, 35% yield without any erosion of enantioselectivity (>99.9% ee) for the major diastereoisomer (See details in the SI). Notably, the sequential oxidative desymmetrization can also be applied to malonic acids bearing tertiary *γ*‐C─H bonds (**S18**), affording the corresponding spirocyclic bilactone (**P18**) in high yield (70%), albeit with reduced enantioselectivity (35% ee). Since the first C─H bond lactonization step dictates enantioselectivity and the subsequent oxidation proceeds from a stereochemically defined lactone intermediate, the observed depletion in ee (relative to the **P6** monoester analog) indicates that the reaction is highly sensitive to subtle structural variations in the substrate. Control experiments involving prolonged stirring of the isolated **P18** bilactone under catalytic conditions ruled out any possible racemization via intramolecular transesterification.

The study was then extended to the oxidation of *α,α−* cycloalkyl malonic acids. Submitting the cyclopentane malonic acid **S19** to the optimized reaction conditions resulted in full recovery of the starting material. Analysis of the crude mixture by ESI‐MS indicated that the lack of reactivity arises from chelation of the two carboxylic acid groups to the metal center (See Figure S2), which prevents H_2_O_2_ activation and subsequent formation of the active metal‐oxo species. The activity was restored by expanding the aliphatic ring of the substrate (**S20**), likely due to suppression of chelation, affording lactone acid **P20** in 51% yield and outstanding enantioselectivity (98% ee). The rigid nature of the [3.2.1]bicyclic structure of **P20** precludes a sequential C−H bond lactonization, leaving a free carboxylic acid available for further chemical elaboration (vide infra). Capitalizing on this finding, we tested different substituted cyclohexane malonic acids (**S21–S23**). Incorporation of a *tert*‐butyl group at the 4‐position of the cyclohexane ring (**S21**) completely inhibited oxidation, likely due to stringent steric demands. Interestingly, when the reaction was performed with catalyst **2^H^**, which features a larger active‐site cavity as recently demonstrated by buried volume analysis [48], formation of lactone **P21** as a single diastereoisomer was observed, albeit in only 19% yield and 41% ee.

Replacing the 4‐*tert*‐butyl substituent with a methyl (**S22**), led to the formation of lactone **P22** in 35% yield when employing doubled loading of catalyst and H_2_O_2_. Complete consumption of the starting material was observed due to competing side reactions or substrate decomposition. The formation of **P22** was observed with a 4:1 diastereomeric ratio, arising from the respective oxidation of the two diastereoisomers bearing *equatorial/axial positioning* of the methyl‐substituent. Nevertheless, the major lactone diastereoisomer restored the high level of enantioselectivity characteristic of these cyclic scaffolds, reaching 99.9% ee. Installation of tertiary centers at the 3‐ and 5‐positions led to the formation of the corresponding bicyclic tertiary lactone in low yield (**P23**, 20%) and excellent enantioselectivity (97% ee). The yield slightly increased to 27% upon doubling catalyst loading, while partial recovery of the starting material suggested product‐induced inhibition of the catalyst. Finally, desymmetrization of the seven‐membered ring dicarboxylic acid **S24** afforded the desired *γ−*lactone (**P24**) as a single diastereomer in 72% yield, with a slight erosion in enantioselectivity (88% ee). No δ‐lactone product was observed, which is attributed to geometric constraints that render the δ‐C─H bonds inaccessible for oxidation.

The free carboxylic acid moiety in these chiral lactones provides an opportunity for further chemical elaboration and expansion of molecular complexity due to its rich and versatile chemistry [58, 59, 60]. The synthetic utility is exemplified by **P20**, which can be directly obtained from commercially and economically available malonic acid **S20** (18$/1 g). **P20** can be scaled up (0.5 g scale) and serves as a cost‐effective chiral starting building block for the construction of more complex scaffolds. Treatment of **P20** with *para*‐toluenesulfonic acid monohydrate provides direct access to decarboxylated product (**P25**), a key intermediate in the synthesis of the natural product Phoslactomycin I‐i, as reported by Kobayashi [61]. Additionally, this lactone is a valuable monomer utilized by Chen for the synthesis of recyclable polymers [62]. This strategy also illustrates that the carboxylic acid moiety can be employed as a removable *α*‐protecting group, as performing the *γ*‐lactonization on the simple cyclohexanecarboxylic acid leads to full substrate consumption but only trace amounts of **P25**, as recently reported [28]. Replacing the carboxylic acid moiety with a heteroatom such as nitrogen opens the possibility for the one‐pot synthesis of chiral amino acids, with no erosion of the enantioselectivity (**P26**, 98% ee). Finally, lactone acid structures can act as directing groups for installing hydroxyl functionalities at the *γ*‐position. Using a standard Fischer esterification procedure, the lactone moiety was opened to give hydroxylated product **P27** in 68% yield with 97% ee, showing moreover that the malonate can be recovered as a chemical handle for further sequential elaboration.

## 3 Conclusions

We report the first example of an asymmetric desymmetrization of readily available malonic monoesters and malonic acids via enantioselective C─H functionalization. Manganese‐catalyzed lactonization delivers valuable chiral *γ*‐ and *δ*‐lactones bearing quaternary stereocenters. The reaction proceeds under mild conditions and short reaction times, employing hydrogen peroxide as a benign terminal oxidant that ensures high atom economy and small loadings of an earth‐abundant metal catalyst, which overall render the process highly attractive for sustainable and scalable synthesis.

This oxidative desymmetrization represents a powerful and broadly applicable platform for the rapid construction of orthogonally manipulable chiral malonic derivatives in a highly versatile manner. The broad availability of the malonic acid substrates and the synthetic relevance of the resulting lactones, which serve as key intermediates in natural product synthesis and materials chemistry, further underscore the potential impact of this methodology. Collectively, the work capitalizes and exemplifies the powerful reach of C─H functionalization methodologies to provide innovative, more sustainable solutions to synthetically relevant problems.

## Conflicts of Interest

The authors declare no conflicts of interest.

## Supporting information




**Supporting File 1**: Materials and Methods describing the preparation of complexes and substrates, characterization, and experimental procedures for the catalytic reactions. Crystallographic data CCDC 2512811 (**P13**), 2512808 (***bis*‐P16**), 2512807 (**P17**), 2512809 (***bis*‐P18**), 2512810 (**P20**), and 2512806 (**P24**) contain the supplementary crystallographic data for this paper. These data can be obtained free of charge from the Cambridge crystallographicdata centre and Fachinformationszentrum Karlsruhe via www.ccdc.cam.ac.uk/structures. NMR Spectra. HPLC traces.

## Data Availability

The data that supports the findings of this study are available in the supplementary material of this article
