# Supplementary material for: Desymmetrization of Malonic Monoesters and Malonic Acids via Enantioselective Catalytic C(sp3)─H Oxidation
Source: Angew Chem Int Ed Engl. 2026 Feb 17;65(13):e1030779. doi: 10.1002/anie.1030779 (PMC13007582; doi:10.1002/anie.1030779)
Supplement: Supplementary file 1 — Supporting File 1: Materials and Methods describing the preparation of complexes and substrates, characterization, and experimental procedures for the catalytic reactions. Crystallographic data CCDC 2512811 (P13), 2512808 ( bis‐P16), 2512807 (P17), 2512809 ( bis‐P18), 2512810 (P20), and 2512806 (P24) contain the supplementary crystallographic data for this paper. These data can be obtained free of charge from the Cambridge crystallographicdata centre and Fachinformationszentrum Karlsruhe via www.ccdc.cam.ac.uk/structures. NMR Spectra. HPLC traces. [file ANIE-65-e1030779-s001.docx]

**Supporting Information**

**Desymmetrization of Malonic Monoesters and Malonic Acids via Enantioselective Catalytic C(sp^3^)−H Oxidation**

Nikos Siakavaras^a^ , Arnau Call,^a,^* Massimo Bietti,^b,^* Miquel Costas^a,^*

*^a^ Institut de Química Computacional i Catàlisi (IQCC) and Departament de Química, Universitat de Girona, Campus Montilivi, Girona E-17071, Catalonia, Spain.*

*^b^ Dipartimento di Scienze e Tecnologie Chimiche, Università “Tor Vergata”, Via della Ricerca*

*Scientifica, 1 I-00133 Rome, Italy.*

*e-mail:* [*arnau.call@udg.edu*](mailto:arnau.call@udg.edu)*; bietti@uniroma2.it, miquel.costas@udg.edu*

**Contents**

| 1. Materials……………………………………………………………………………... | 3 |
| --- | --- |
| 1. Methods……………………………………………………………………………… | 3 |
| 1. Instrumentation……………………………………………………………………… | 3 |
| 1. Synthesis of catalysts………………………………………………………………... | 4 |
| 1. Synthesis of substrates………………………………………………………………. | 4 |
| 1. Reaction optimization for *δ*-C**-**H lactonization of **S10**…………………………………... | 20 |
| 1. Catalytic studies……………………………………………………………………............... | 21 |
| 7.1. General oxidation protocol…………………………………………………………...... | 21 |
| 7.2. General oxidation protocol for product isolation………………………………………. | 21 |
| 7.3. Characterization of isolated products…………………………………………............... | 22 |
| 7.4. Product elaboration…………………………………………………………………….. | 30 |
| 1. Sequential lactonization of **S17** by double addition of catalyst……………………............... | 32 |
| 1. Catalyst inhibition by cyclopentane dicarboxylic acid **S19**…………………………………. | 33 |
| 1. Solid state structures by X-ray diffraction analysis…………………………………………. | 34 |
| 10.1. Solid state structure of **P13**……………………………………………………………. | 34 |
| 10.2. Solid state structure of ***bis-*P16^syn-sym^**………………………………………………….. | 36 |
| 10.3. Solid state structure of **P17**.…………………………………………………………… | 38 |
| 10.4. Solid state structure of ***bis-*P18**………………………………………………………... | 40 |
| 10.5. Solid state structure of **P20**……………………………………………………………. | 42 |
| 10.6. Solid state structure of **P24**……………………………………………………………. | 44 |
| 1. ^1^H and ^13^C-NMR of the substrates…………………………………………………………... | 46 |
| 1. ^1^H and ^13^C-NMR of the isolated lactones…………………………………………................ | 80 |
| 1. ^1^H and ^13^C-NMR of the product elaboration………………………………………………… | 112 |
| 1. ^1^H and ^13^C-NMR of the derivatized lactones………………………………………………... | 115 |
| 1. SFC and GC traces…………………………………………………………………………... | 127 |
| 1. References…………………………………………………………………………................ | 173 |
|  |  |
|  |  |

**1. Materials**

Reagents and solvents used were of commercially available reagent quality unless stated otherwise. 2,2,2-Trifluoroethanol (TFE) and 1,1,1,3,3,3-hexafluoro-2-propanol (HFIP) were purchased from Fluorochem while the other solvents were purchased from SDS and Scharlab. Anhydrous solvents were purified and dried by passing through an activated alumina purification system (M-Braun SPS-800). Hydrogen peroxide solutions employed in the oxidation reactions were prepared by diluting commercially available hydrogen peroxide (50% H_2_O_2_ solution in water, Aldrich).

**2. Methods**

Unless otherwise indicated, all reactions for the preparation of substrates and catalysts were performed in standard dry glassware under a nitrogen atmosphere. Reported concentrations refer to solution volume at room temperature. Organic solvents were removed under reduced pressure using house vacuum (40 torr) at 30 ºC. Column chromatography was performed on silica gel (SilicaFlash P60, 230-400, mesh SiliCycle). Thin-layer chromatography (TLC) was used for reaction monitoring and product detection, employing TLC plates from Scharlau. Plates were visualized by exposure to UV (λ = 254 nm) or by staining with bromocresol green or phosphomolydbic acid.

**3. Instrumentation**

Oxidation products were identified by comparison of their GC retention times and GC/MS data with those of authentic compounds, and by ^1^H and ^13^C-NMR analyses. X-ray diffraction analysis was carried out on a BRUKER SMART APEX CCD diffractometer using graphite-monochromated MoKα radiation (λ = 0.71073 Å) from an X-ray tube. NMR spectra were recorded on BrukerDPX300 and DPX400 spectrometers using standard conditions. Electrospray ionization mass spectrometry (ESI-MS) experiments were performed on a Bruker Daltonics Esquire 3000 Spectrometer using a 1 mM solution of the compound analyzed. High-resolution mass spectra (HRMS) were recorded on a Bruker MicroTOF-QII (Q-TOF) instrument equipped with an ESI source at the Serveis Tècnics of the Universitat de Girona. Samples were introduced into the ion source through a syringe pump and were externally calibrated using sodium formate. Optical rotations were measured at 20 ºC using a Jasco P2000 iRM-800 polarimeter. Concentration is expressed in g/100 mL, and measurements were performed in a 10 cm long cell with a 1 mL capacity. Chromatographic analyses were performed on an Agilent GC-7820-A chromatograph using an HP5 column (30 m), while enantiomeric resolution using HP-Chiral20B and J&W CYCLOSIL-B columns, as well as supercritical fluid chromatography (SFC), was carried out on an Agilent 1260 Infinity II SFC System using CHIRALPAK IG-3 column

**4. Synthesis of catalysts**

The complexes (*S,S*)-Mn(OTf)_2_(pdp)^[1]^, (S,S)-Mn(OTf)_2_(^TIPS^pdp)^[2]^, (*S,S*)-Mn(^Hbz^bpeb)^[3]^, (*S,S*)-Mn^iPr^(bpeb) and (*S,S*)-Mn^CF3^(bpeb)^[4]^ were prepared according to the reported procedures.

**5. Synthesis of substrates**

Substrate **S20** is commercially available was purchased from Merck, whereas the remaining substrates were prepared according to the procedures described in this section.

**Malonic acid monoesters synthesis: protocol A**

For malonic acid monoester synthesis, **protocol A** was followed as a slight modification of a reported procedure. ^[5]^ A round-bottom flask equipped with a septum and maintained under nitrogen was charged with dry THF and the diester malonate **A** (2 g, 1 equiv.). The reaction mixture was cooled to 0 °C, and NaH (2.1 equiv) was slowly added in portions. The mixture was stirred for 30 minutes at 0 °C, after which the corresponding alkyl halide (2.1 equiv.) was added slowly. The reaction was stirred overnight, allowing the temperature to rise to room temperature. Reaction progress was monitored by GC, and additional alkyl halide was added when starting material or monoalkylated intermediate remained. After completion, the reaction was quenched in an ice bath under a nitrogen purge by the addition of H₂O and then diluted with dichloromethane. The organic layer was separated, and the aqueous layer was extracted twice more with dichloromethane. The combined organic layers were washed once with H_2_O and with brine, dried over anhydrous magnesium sulfate (MgSO_4_), filtered, and evaporated to dryness. To the crude residue, without further purification, NaOH (0.9 equiv) and methanol were added as solvent, and the mixture was stirred overnight at 35°C. The reaction mixture was then acidified to pH~1 with HCl 2M and extracted three times with dichloromethane. The combined organic layers were washed twice with H_2_O, dried over MgSO_4_, filtered, and evaporated to dryness. The desired monohydrolyzed product was purified by flash chromatography on silica gel.

**Malonic acid monoesters synthesis: protocol B**

For the synthesis of malonic acid monoesters, **protocol B**, the alkylation step was carried out following a slight modification of a reported procedure.^[6]^ A round-bottom flask equipped with a septum and maintained under nitrogen was charged with dry DMF and the Meldrum’s acid **B** (2 g, 1 equiv). K_2_CO_3_ (4 equiv.) was then added in small portions to the reaction at room temperature, and the mixture was stirred for 30 minutes. After that, the corresponding alkyl halide (4 equiv.) was slowly added. Upon fully conversion of the starting material **B**, the solvent was evaporated under reduced pressure, and the crude mixture was purified by flash chromatography on silica gel to obtain the desired dialkylated Meldrum’s intermediate.

For the ring-opening step, malonic acids monoesters were prepared following the reported procedure.^[7]^ To a solution of the dialkylated intermediate in a 5:1 mixture of R_1_OH:THF, KOH (2.5 M) was added, and the reaction was stirred at room temperature overnight. The mixture was then acidified to pH~1 with 2 M HCl and extracted three times with dichloromethane. The organic layers were combined, washed with twice with H_2_O, dried over anhydrous MgSO_4_, filtered and evaporated to dryness. The desired monohydrolyzed product was purified after purification by flash chromatography on silica gel.

**2-ethyl-2-(methoxycarbonyl)butanoic acid (S1).** Following the general conditions of malonic *acid monoesters synthesis protocol A*, iodoethane was used as the alkyl halide. The crude product was purified by flash chromatography on silica gel using hexane:EtOAc (10:1) to afford **S1** as a white solid (1.14 g, 6.54 mmol, 43% total yield over two steps). ^1^H-NMR (400 MHz, CDCl_3_) δ, ppm: 3.80 (s, 3H), 2.08 – 1.86 (m, 4H), 0.85 (t, *J* = 7.5 Hz, 6H). ^13^C-NMR (101 MHz, CDCl_3_) δ, ppm: 175.2, 171.0, 59.1, 53.0, 28.0, 9.3. HRMS (ESI-MS) *m/z* calculated for C_8_H_14_O_4_ [M+Η]^+^ 175.0965, found 175.0965.

**2-(ethoxycarbonyl)-2-ethylbutanoic acid (S2)**: Following the general conditions of malonic *acid monoesters synthesis protocol A*, iodoethane was used as the alkyl halide. The crude product was purified by flash chromatography on silica gel using hexane:EtOAc (10:1) to afford **S2** as colorless liquid (942 mg, 5.21 mmol, 43% total yield over two steps). Spectroscopic data matched with reported values.^[8]^ ^1^H-NMR (400 MHz, CDCl_3_) δ, ppm: 11.60 (br, 1H), 4.29 (q, *J* = 7.2 Hz, 2H), 2.03 (dq, *J* = 14.8, 7.5 Hz, 2H), 1.91 (dq, *J* = 14.8, 7.5 Hz, 2H), 1.32 (t, *J* = 7.2 Hz, 3H), 0.86 (t, *J* = 7.6 Hz, 6H). ^13^C-NMR (101 MHz, CDCl_3_) δ, ppm: 175.7, 174.3, 62.4, 59.2, 28.9, 14.2, 9.4. HRMS (ESI-MS) *m/z* calculated for C_9_H_16_O_4_ [M+Na]^+^ 211.0941, found 211.0942.

**5,5-diethyl-2,2-dimethyl-1,3-dioxane-4,6-dione (S3a):** Following the general conditions of malonic *acid monoesters synthesis protocol B*, iodoethane was used as the alkyl halide. The crude product was purified by flash chromatography on silica gel using hexane:EtOAc (30:1) to afford **S3a** as a white solid (1.76 g, 8.8 mmol, 63% yield). ^1^H-NMR (400 MHz, CDCl_3_) δ, ppm: 2.02 (q, *J* = 7.5 Hz, 4H), 1.70 (s, 6H), 0.93 (t, *J* = 7.5 Hz, 4H). ^13^C-NMR (101 MHz, CDCl_3_) δ, ppm: 169.5, 105.5, 56.4, 32.5, 29.9, 10.3.

**2-ethyl-2-((2,2,2-trifluoroethoxy)carbonyl)butanoic acid (S3)**: Following the general conditions of malonic *acid monoesters synthesis protocol B*, starting from **S3a** (700 mg) and using TFE:THF (5:1) as the solvent, the crude product was purified by flash chromatography on silica gel using hexane:EtOAc (10:1) to afford **S3** as colorless liquid (412 mg, 1.70 mmol, 49% yield). ^1^H-NMR (400 MHz, CDCl_3_) δ, ppm: 4.55 (q, *J* = 8.3 Hz, 2H), 2.00 (q, *J* = 7.6 Hz, 4H), 0.88 (t, *J* = 7.6 Hz, 6H). ^13^C-NMR (101 MHz, CDCl_3_) δ, ppm: 175.7, 170.8, 123.7 (q, *J* = 276.74 Hz), 60.9 (q, *J* = 37.0 Hz) 58.9, 25.9, 8.6. ^19^F-NMR (377 MHz, CDCl_3_) δ, ppm: -74.7. HRMS (ESI-MS) *m/z* calculated for C_9_H_13_F_3_O_4_ [M+H]^+^ 243.0839, found 243.0840.

**2-(methoxycarbonyl)-2-propylpentanoic acid (S4).** Following the general conditions of malonic *acid monoesters synthesis protocol A*, 1-iodopropane was used as the alkyl halide. The crude product was purified by flash chromatography on silica gel using hexane:EtOAc (10:1) to afford **S4** as a white solid (1.42 g, 7.03 mmol, 46% total yield over two steps). ^1^H-NMR (400 MHz, CDCl_3_) δ, ppm: 3.81 (s, 3H), 1.96 (ddd, *J* = 13.8, 12.1, 4.8 Hz, 2H), 1.83 (ddd, *J* = 13.8, 12.0, 4.9 Hz, 2H), 1.33 – 1.08 (m, 4H), 0.90 (t, *J* = 7.3 Hz, 6H). ^13^C-NMR (101 MHz, CDCl_3_) δ, ppm: 176.4, 174.4, 58.0, 53.2, 38.4, 18.5, 14.2. HRMS (ESI-MS) *m/z* calculated for C_10_H_18_O_4_ [M+Η]^+^ 203.1278, found 203.1276.

 **2,2-dimethyl-5,5-diphenethyl-1,3-dioxane-4,6-dione (S5a):** Following the general conditions of malonic *acid monoesters synthesis protocol B*, 2-phenylethyl bromide was used as the alkyl halide. The crude product was purified by flash chromatography on silica gel using hexane:EtOAc (100:1) to afford **S5a** as a white solid (807 mg, 2.29 mmol, 17% yield). ^1^H-NMR (400 MHz, CDCl_3_) δ, ppm: 7.29 (t, *J* = 7.3 Hz, 4H), 7.21 (t, *J* = 7.3 Hz, 2H), 7.16 (d, *J* = 6.8 Hz, 4H), 2.65 – 2.61 (m, 4H), 2.35 – 2.31 (m, 4H), 1.81 (s, 6H). ^13^C-NMR (101 MHz, CDCl_3_) δ, ppm: 169.2, 140.0, 128.8, 128.5, 126.7, 105.9, 54.7, 41.1, 32.1, 30.0.

**2-(methoxycarbonyl)-2-phenethyl-4-phenylbutanoic acid (S5):** Following the general conditions of malonic *acid monoesters synthesis protocol B*, starting from **S5a** (400 mg) and using MeOH:THF (5:1) as the solvent, the crude product was purified by flash chromatography on silica gel using hexane:EtOAc (10:1) to afford **S5** as a white solid (190 mg, 0.58 mmol, 51% yield). ^1^H-NMR (400 MHz, CDCl_3_) δ, ppm: 7.29 (t, *J* = 7.2 Hz, 4H), 7.23 – 7.17 (m, 6H), 3.72 (s, 3H), 2.67 – 2.52 (m, 4H), 2.40 – 2.26 (m, 4H). ^13^C-NMR (101 MHz, CDCl_3_) δ, ppm: 175.5, 173.8, 140.8, 128.6, 128.5, 126.4, 57.7, 53.0, 36.6, 31.2. HRMS (ESI-MS) *m/z* calculated for C_20_H_22_O_4_ [M+Η]^+^ 327.1591, found 327.1586.

**5,5-diisobutyl-2,2-dimethyl-1,3-dioxane-4,6-dione (S6a):** Following the general conditions of malonic *acid monoesters synthesis protocol B*, 1-iodo-2-methylpropane was used as the alkyl halide. The crude product was purified by flash chromatography on silica gel using hexane:EtOAc (50:1) to afford **S6a** as a white solid (1.12 g, 4.38 mmol, 32% yield). ^1^H-NMR (400 MHz, CDCl_3_) δ, ppm: 1.96 (d, *J* = 6.1 Hz, 4H), 1.77 (s, 6H), 1.67 (hept, *J* = 6.2 Hz, 2H), 0.91 (d, *J* = 6.6 Hz, 12H). ^13^C-NMR (101 MHz, CDCl_3_) δ, ppm: 169.4, 105.8, 52.6, 49.5, 30.0, 25.7, 23.9.

**2-isobutyl-2-(methoxycarbonyl)-4-methylpentanoic acid (S6):** Following the general conditions of malonic *acid monoesters synthesis protocol B*, starting from **S6a** (750 mg) and using MeOH:THF (5:1) as the solvent, the crude product was purified by flash chromatography on silica gel using hexane:EtOAc (10:1) to afford **S6** as a white solid (526 mg, 2.29 mmol, 78% yield). ^1^H NMR (400 MHz, CDCl_3_) δ, ppm: 3.83 (s, 3H), 2.01 (dd, *J* = 13.8, 6.5 Hz, 2H), 1.79 (dd, *J* = 13.8, 6.5 Hz, 2H), 1.53 (hept, 6.5 Hz, 2H), 0.87 (d, *J* = 6.6 Hz, 6H), 0.82 (d, *J* = 6.6 Hz, 6H). ^13^C NMR (101 MHz, CDCl_3_) δ, ppm: 179.7, 173.6, 55.6, 53.2, 48.3, 25.8, 23.3, 22.9. HRMS (ESI-MS) *m/z* calculated for C_12_H_22_O_4_ [M+Η]^+^ 231.1591, found 231.1585.

**5,5-dibutyl-2,2-dimethyl-1,3-dioxane-4,6-dione (S7a):** Following the general conditions of malonic *acid monoesters synthesis protocol B*, 1-iodobutane was used as the alkyl halide. The crude product was purified by flash chromatography on silica gel using hexane:EtOAc (100:1) to afford **S7a** as a white solid (1.04 g, 4.0 mmol, 29% yield). ^1^H-NMR (400 MHz, CDCl_3_) δ, ppm: 2.01 – 1.97 (m, 4H), 1.73 (s, 6H), 1.28 (tt, *J* = 14.3, 7.0 Hz, 8H), 0.88 (t, *J* = 7.0 Hz, 6H). ^13^C-NMR (101 MHz, CDCl_3_) δ, ppm: 169.8, 105.6, 54.9, 39.4, 30.0, 28.0, 22.7, 13.8.

**2-butyl-2-(methoxycarbonyl)hexanoic acid (S7):** Following the general conditions of malonic *acid monoesters synthesis protocol B*, starting from **S7a** (510 mg) and using MeOH:THF (5:1) as the solvent, the crude product was purified by flash chromatography on silica gel using hexane:EtOAc (10:1) to afford **S7** as a white solid (360 mg, 1.5 mmol, 79% yield). ^1^H- NMR (400 MHz, CDCl_3_) δ, ppm: 10.10 (br, 1H) 3.75 (s, 3H), 1.96 – 1.81 (m, 4H), 1.29 (h, *J* = 7.3 Hz, 4H), 1.19 – 1.09 (m, 4H), 0.87 (t, *J* = 7.3 Hz, 6H). ^13^C-NMR (101 MHz, CDCl_3_) δ, ppm: 176.8, 174.1, 57.9, 52.8, 33.9, 26.7, 22.9, 13.9. HRMS (ESI-MS) *m/z* calculated for C_12_H_22_O_4_ [M+Η]^+^ 231.1591, found 231.1589.

**5,5-diisopentyl-2,2-dimethyl-1,3-dioxane-4,6-dione (S8a):** Following the general conditions of malonic *acid monoesters synthesis protocol B*, isopentyl bromide was used as the alkyl halide. The crude product was purified by flash chromatography on silica gel using hexane:EtOAc (100:1) to afford **S8a** as a white solid (900 mg, 3.1 mmol, 23% yield). Spectroscopic data matched with previous reported ones.^[7]^ ^1^H-NMR (400 MHz, CDCl_3_) δ, ppm: 2.03 – 1.98 (m, 4H), 1.73 (s, 6H), 1.50 (hept, *J* = 6.6 Hz, 2H), 1.29 – 1.13 (m, 4H), 0.87 (d, *J* = 6.6 Hz, 12H). ^13^C-NMR (101 MHz, CDCl_3_) δ, ppm: 169.7, 105.6, 54.9, 37.7, 34.7, 30.0, 28.2, 22.4.

**2-isopentyl-2-(methoxycarbonyl)-5-methylhexanoic acid (S8)**:

Following the general conditions of malonic *acid monoesters synthesis protocol B*, starting from **S8a** (800 mg) and using MeOH:THF (5:1) as the solvent, the crude product was purified by flash chromatography on silica gel using hexane:EtOAc (10:1) to afford **S8** as a white solid (567 mg, 2.2 mmol, 78% yield). Spectroscopic data matched with previous reported ones.^[7]^ ^1^H-NMR (400 MHz, CDCl_3_) δ, ppm: 11.45 (br, 1H), 3.82 (s, 3H), 1.99 (tdd, *J* = 12.8, 4.7, 1.9 Hz, 2H), 1.86 (td, *J* = 13.2, 4.5 Hz, 2H), 1.49 (hept, *J* = 6.6 Hz, 2H), 1.21 – 1.05 (m, 2H), 1.02 – 0.90 (m, 2H), 0.86 (d, *J* = 6.6 Hz, 12H). ^13^C-NMR (101 MHz, CDCl_3_) δ, ppm: 176.5, 174.2, 57.9, 53.2, 34.1, 33.9, 28.23 22.5, 22.4. HRMS (ESI-MS) *m/z* calculated for C_14_H_26_O_4_ [M+Η]^+^ 259.1904, found 259.1902.

 **2,2-dimethyl-5,5-bis(3-phenylpropyl)-1,3-dioxane-4,6-dione (S9a):** Following the general conditions of malonic *acid monoesters synthesis protocol B*, (3-bromopropyl)benzene was used as the alkyl halide. The crude product was purified by flash chromatography on silica gel using hexane:EtOAc (100:1) to afford **S9a** as a white solid (1040 mg, 2.74 mmol, 20% yield). ^1^H- NMR (400 MHz, CDCl_3_) δ, ppm: 7.27 (t, *J* = 7.3 Hz, 4H), 7.23 – 7.15 (m, 2H), 7.12 (d, *J* = 6.8 Hz, 4H), 2.60 (t, *J* = 7.6 Hz, 4H), 2.05 – 2.00 (m, 4H), 1.67 (s, 6H), 1.65 – 1.58 (m, 4H). ^13^C-NMR (101 MHz, CDCl_3_) δ, ppm: 169.4, 141.0, 128.6, 128.4, 126.2, 105.7, 54.6, 38.9, 35.7, 29.9, 27.3.

**2-(methoxycarbonyl)-5-phenyl-2-(3-phenylpropyl)pentanoic acid (S9):**

Following the general conditions of malonic *acid monoesters synthesis protocol B*, starting from **S9a** (900 mg) and using MeOH:THF (5:1) as the solvent, the crude product was purified by flash chromatography on silica gel using hexane:EtOAc (10:1) to afford **S9** as a white solid (520 mg, 1.46 mmol, 62% yield). ^1^H-NMR (400 MHz, CDCl_3_) δ, ppm: 7.27 (t, *J* = 7.3 Hz, 4H), 7.18 (t, *J* = 7.3 Hz, 0H), 7.12 (d, *J* = 6.8 Hz, 4H), 3.72 (s, 3H), 2.58 (qdd, *J* = 14.1, 8.5, 6.4 Hz, 4H), 2.01 (td, *J* = 13.1, 4.6 Hz, 2H), 1.92 – 1.83 (m, 2H), 1.61 – 1.50 (m, 2H), 1.46 – 1.36 (m, 2H). ^13^C-NMR (101 MHz, CDCl_3_) δ, ppm: 175.8, 173.8, 141.5, 128.5, 128.4, 126.1, 57.6, 53.2, 35.7, 35.4, 26.7. HRMS (ESI-MS) *m/z* calculated for C_22_H_26_O_4_ [M+Η]^+^ 355.1904, found 355.1909.

**5-phenyl-2-(3-phenylpropyl)-2-((2,2,2-trifluoroethoxy)carbonyl)pentanoic acid (S10):**

Following the general conditions of malonic *acid monoesters synthesis protocol B*, starting from **S9a** (500 mg) and using TFE:THF (5:1) as the solvent, the crude product was purified by flash chromatography on silica gel using hexane:EtOAc (10:1) to afford **S10** as a colorless liquid (378 mg, 0.89 mmol, 68% yield). ^1^H-NMR (400 MHz, CDCl_3_) δ, ppm: 10.01 (br, 1H), 7.31 (t, *J* = 7.4 Hz, 4H), 7.22 (t, *J* = 7.3 Hz, 2H), 7.16 (d, *J* = 6.9 Hz, 4H), 4.51 (q, *J* = 8.3 Hz, 2H), 2.63 (t, *J* = 7.5 Hz, 4H), 1.99 (t, *J* = 8.5 Hz, 4H), 1.58 – 1.42 (m, 4H). ^13^C-NMR (101 MHz, CDCl_3_) δ, ppm 176.7, 170.2, 141.5, 128.5, 128.4, 126.1,122.8 (q, *J* = 277.5 Hz), 60.8 (q, *J* = 36.9 Hz), 35.9, 32.4, 25.9. ^19^F-NMR (377 MHz, CDCl_3_) δ, ppm: -74.6. HRMS (ESI-MS) *m/z* calculated for C_23_H_25_F_3_O_4_ [M+NΗ_4_]^+^ 440.2043, found 440.2045.

**1-(methoxycarbonyl) cyclopentane-1-carboxylic acid (S11).** Following the general conditions of malonic *acid monoesters synthesis protocol A*, 1,4-dibromobutane was used as the alkyl halide. The crude product was purified by flash chromatography on silica gel using hexane:EtOAc (10:1) to afford **S11** as a white solid (1.65 g, 9.59 mmol, 63% total yield over two steps). ^1^H-NMR (400 MHz, CDCl_3_) δ, ppm: 10.69 (br, 1H), 3.75 (s, 3H), 2.24 – 2.21 (m, 4H), 1.74 – 1.70 (m, 4H). ^13^C-NMR (101 MHz, CDCl_3_) δ, ppm: 178.6, 173.0, 60.3, 52.9, 34.9, 25.7. HRMS (ESI-MS) *m/z* calculated for C_8_H_12_O_4_ [M+Η]^+^ 173.0808, found 173.0805.

**1-(methoxycarbonyl)cyclohexane-1-carboxylic acid (S12).** Following the general conditions of malonic *acid monoesters synthesis protocol A*, 1,5-dibromopentane was used as the alkyl halide. The crude product was purified by flash chromatography on silica gel using hexane:EtOAc (10:1) to afford **S12** as a white solid (950 mg, 5.11 mmol, 34% total yield over two steps). ^1^H-NMR (400 MHz, CDCl_3_) δ, ppm: 3.75 (s, 3H), 2.08 – 1.99 (m, 4H), 1.54 (hex, *J* = 5.8 Hz, 4H), 1.45 (q, *J* = 5.6 Hz, 2H). ^13^C-NMR (101 MHz, CDCl_3_) δ, ppm: 177.4, 172.1, 55.1, 52.8, 31.4, 25.2, 22.8. HRMS (ESI-MS) *m/z* calculated for C_9_H_14_O_4_ [M+Η]^+^ 187.0966, found 187.0965.


 **1-(methoxycarbonyl)cycloheptane-1-carboxylic acid (S13):** Substrate **S13** was prepared according to reported procedure.^[9]^ Under a N_2_ atmosphere, diisopropylamine (7.3 mL, 52.7 mmol, 2.5 equiv.) was dissolved in anhydrous THF (60 mL). The mixture was cooled to -78°C and *n*-BuLi (21.1 mL, 2.5 M in hexane, 52.7 mmol, 2.5 equiv.) was added dropwise. After the addition was complete, 1 equiv. of cycloheptanecarboxylic acid (3 g, 21.1 mmol) was added and the mixture was allowed to warm to room temperature, then heated to reflux for 4 h before 1 equiv. of methyl chloroformate (1.60 mL, 21.1 mmol) was added dropwise. The reaction was stirred at room temperature overnight. It was then quenched with saturated NH_4_Cl (aq) (10 mL), acidified with 2 M HCl (20 mL), and the product was extracted with Et_2_O (3 x 15 mL). The combined organic phases were dried over anhydrous MgSO_4_, filtered and concentrated under reduced pressure. The product was purified by flash chromatography on silica using hexane:EtOAc (10:1) to afford **S13** as a white solid (3.30 g, 16.5 mmol, 78% yield). ^1^H-NMR (400 MHz, CDCl_3_) δ, ppm: 3.74 (s, 3H), 2.15 – 2.13 (m, 4H), 1.60 – 1.55 (m, 8H). ^13^C-NMR (101 MHz, CDCl_3_) δ, ppm: 177.7, 173.2, 57.7, 52.8, 33.9, 30.0, 24.0. HRMS (ESI-MS) *m/z* calculated for C_10_H_16_O_4_ [M+Η]^+^ 201.1121, found 201.1120.


**(methoxymethylene)cyclooctane (S14a)**: Substrate **S14a** was prepared according to reported procedure.^[10]^ To a suspension of (methoxymethyl)triphenylphosphonium chloride (6.2 g, 18.25 mmol, 1.15 equiv.) in anhydrous THF (80 mL) under N_2_ at 0 ºC (water-ice bath), potassium *tert*-butoxide (2.1 g, 19.0 mmol, 1.2 equiv.) was added as a solid as small portions. After the additions, the mixture immediately turned orange, and it was stirred for 60 min at 0 ºC. After that, cyclooctenone (15.87 mmol, 1 equiv., 1 M in THF) was added, and the resulting mixture was stirred for 18 h while warming to room temperature. The reaction was quenched with Et_2_O and saturated aqueous ammonium chloride. The product was extracted twice with Et_2_O, and the combined organic layers were washed once with water, once with brine, and dried over anhydrous MgSO_4_. The solvent was removed under reduced pressure, and the product was purified by flash chromatography on silica using pentane to afford **S14a** as a colorless liquid (1.17 g, 7.59 mmol, 48% yield). Spectroscopic data matched with previous reported ones. ^1^H-NMR (400 MHz, CDCl_3_) δ, ppm: 5.77 (s, 1H), 3.54 (s, 6H), 2.27 – 2.14 (m, 3H), 2.07 – 1.95 (m, 4H), 1.68 – 1.55 (m, 4H), 1.55 – 1.44 (m, 6H).

 **cyclooctanecarbaldehyde (S14b)**: Substrate **S14b** was prepared according to reported procedure.^[11]^ To enol ether **S14a** (1.17 g, 7.59 mmol) was added 2 M HCl (in THF:H_2_O 1:1) and the reaction was stirred at 90 °C for 2 h. The reaction was allowed to cool to room temperature and the product extracted with Et_2_O (3 × 20 mL). The combined organic layers were dried over anhydrous MgSO_4_, filtered and the solvent removed under reduced pressure to afford **S14b** as a colorless oil (1.06 g, 7.57 mmol, 99% yield). Spectroscopic data matched with previous reported ones. ^1^H-NMR (400 MHz, CDCl_3_) δ, ppm: 9.61, ppm: (s, 1H), 2.40 – 2.33 (m, 1H), 1.98 – 1.92 (m, 2H), 1.78 – 1.65 (m, 2H), 1.62 – 1.47 (m, 10H).

 **cyclooctanecarboxylic acid** (**S14c):** Substrate **S14c** was prepared according to reported procedure.^[12]^ Aldehyde **S14b** (500 mg, 3.57 mmol, 1 equiv.) was dissolved in acetone (15 mL), and a freshly prepared Jones reagent was added dropwise, until the color of the solution remains orange for 15 min. The excess of Jones reagent was quenched with isopropanol (5 mL). The mixture was filtered through celite, acidified with 2M HCl (7 mL) and the product was extracted with Et_2_O (3 x 15 mL). The combined organic phases were dried over anhydrous MgSO_4_, filtered and concentrated under reduced pressure. The product was purified by flash chromatography on silica gel using hexane:EtOAc (8:1) to afford **S14c** as a white solid (368 mg, 2.35 mmol, 66% yield). Spectroscopic data matched with previous reported ones.^[13]^ ^1^H-NMR (400 MHz, CDCl_3_) δ, ppm: 2.58 – 2.51 (m,1H), 2.02 – 1.86 (m, 2H), 1.76 – 1.68 (m, 4H), 1.64 – 1.45 (m, 8H).

**1-(methoxycarbonyl)cyclooctane-1-carboxylic acid (S14):** Substrate **S14** was prepared according to a analogous procedure for **S13**. Under a N_2_ atmosphere, diisopropylamine (0.84 mL, 6.0 mmol, 2.5 equiv.) was dissolved in anhydrous THF (20 mL). The mixture was cooled to -78^°^C and 2.4 mL of *n*-BuLi (2.5 M in hexane, 6.0 mmol, 2.5 equiv.) were added dropwise. When the addition was completed, **S14c** (368 mg, 2.39 mmol, 1 equiv.) was added, and the mixture was stirred while warming to room temperature, then under reflux at 60 ^o^C for 4 h before methyl chloroformate (0.19 mL, 2.39 mmol, 1 equiv.) was added dropwise. The reaction was stirred at room temperature overnight. After that, it was quenched with saturated NH_4_Cl (aq) (5 mL), acidified with 2 M HCl (7 mL), and the product was extracted with Et_2_O (3 x 15 mL). The combined organic phases were dried over anhydrous MgSO_4_, filtered, and concentrated under reduced pressure. The product was purified by flash chromatography on silica gel using hexane:EtOAc (10:1) to afford **S14** as a white solid (324 mg, 1.5 mmol, 64% yield). ^1^H-NMR (400 MHz, CDCl_3_) δ, ppm: 3.74 (s, 3H), 2.16 (br, 4H), 1.55 (br, 10H). ^13^C-NMR (101 MHz, CDCl_3_) δ, ppm: 177.5, 172.7, 57.9, 52.7, 29.0, 28.2, 25.0, 22.81. HRMS (ESI-MS) *m/z* calculated for C_11_H_18_O_4_ [M+Η]^+^ 215.1278, found 215.1280.

**2,2-diethylmalonic acid (S15):** Following the general conditions of malonic *acid monoesters synthesis protocol B*, starting from **S3a** and using H_2_O:THF (5:1) as the solvent, the crude product was purified by flash chromatography on silica gel using hexane:EtOAc (5:1) to afford **S15** as a white solid (410 mg, 2.56 mmol, 64% yield). ^1^H-NMR (400 MHz, CDCl_3_) δ, ppm: 2.02 (q, *J* = 7.5 Hz, 4H), 0.92 (t, *J* = 7.5 Hz, 6H). ^13^C-NMR (101 MHz, CDCl_3_) δ, ppm: 176.6, 59.1, 28.7, 9.4. HRMS (ESI-MS) *m/z* calculated for C_7_H_11_O_4_ [M-Η]^-^ 159.0663, found 159.0660.


**2,2-dipropylmalonic acid (S16):** Under a N_2_ atmosphere, **S4** (606 mg, 3.0 mmol, 1 equiv.) was dissolved in anhydrous THF (20 mL). The mixture was cooled to 0 ^o^C and LiAlH_4_ (360 mg, 9.0 mmol, 3 equiv.) was added in portions as a solid. The resulting mixture was left stirring until it reached room temperature. After full conversion of the starting ester the mixture was quenched with 2M NaOH and the product was extracted with EtOAc (3 x 15 mL). The combined organic phases were dried over anhydrous MgSO_4_, filtered and concentrated under reduced pressure to give in quantitative yield the intermediate diol product **S16a**. The diol was then dissolved in acetone (15 mL), and freshly prepared Jones reagent was added dropwise until the color of the solution remained orange for 15 minutes. The excess of Jones reagent quenched by addition of isopropanol (5 mL). The mixture was filtered through celite, acidified with 2 M HCl (7 mL), and extracted with EtOAc (3 x 15 mL). The combined organic phases were dried over anhydrous MgSO_4_, filtered, and concentrated under reduced pressure. The product was purified by flash chromatography on silica gel using hexane:EtOAc (10:1) to afford **S16** as a white solid (320 mg, 1.70 mmol, 57% total yield over two steps). Spectroscopic data matched with previous reported ones.^[14]^ ^1^H-NMR (400 MHz, CD_3_OD) δ, ppm: 1.86 – 1.78 (m, 4H), 1.30 – 1.15 (m, 4H), 0.93 (t, *J* = 7.3 Hz, 6H). ^13^C-NMR (101 MHz, CD_3_OD) δ, ppm: 175.8, 58.6, 36.4, 18.7, 14.7. HRMS (ESI-MS) *m/z* calculated for C_9_H_16_O_4_ [M+Η]^+^ 189.1121, found 189.1121.

 **2,2-diphenethylmalonic acid (S17):** Following the general conditions of malonic *acid monoesters synthesis protocol B*, starting from **S5a** (300 mg) and using H_2_O:MeOH (5:1) as the solvent, the crude product was purified by flash chromatography on silica gel using hexane:EtOAc (5:1) to afford **S17** as a white solid (200 mg, 0.64 mmol, 75% yield). ^1^H-NMR (400 MHz, CD_3_OD) δ, ppm: 7.90 – 7.24 (m, 4H), 7.18 – 7.14 (m, 6H), 2.58 – 2.54 (m, 4H), 2.22 – 2.17 (m, 4H). ^13^C-NMR (101 MHz, CD_3_OD) δ, ppm: 175.1, 143.1, 129.5, 129.3, 127.0, 58.9, 36.3, 32.0. HRMS (ESI-MS) *m/z* calculated for C_19_H_20_O_4_ [M-Η]^-^ 311.1289, found 311.1290.

 **2,2-diisobutylmalonic acid (S18):** Following the general conditions of malonic *acid monoesters synthesis protocol B*, starting from **S6a** (500 mg) and using H_2_O:MeOH (5:1) as the solvent, the crude product was purified by flash chromatography on silica gel using hexane:EtOAc (5:1) to afford **S18** as a white solid (360 mg, 1,67 mmol, 85% yield). ^1^H-NMR (400 MHz, CD_3_OD) δ, ppm: 1.87 (d, *J* = 6.5 Hz, 4H), 1.62 (hept, *J* = 6.5 Hz, 2H), 0.90 (d, *J* = 6.5 Hz, 12H). ^13^C-NMR (101 MHz, CD_3_OD) δ, ppm: 177.4, 56.8, 44.9, 26.0, 23.9. HRMS (ESI-MS) *m/z* calculated for C_11_H_20_O_4_ [M-Η]^-^ 215.1289, found 215.1288.


**cyclopentane-1,1-dicarboxylic acid (S19)**: To a solution of **S11** (172 mg, 1.0 mmol) in MeOH/H_2_O (1:1, 40 mL), NaOH (800 mg, 20.0 mmol, 20.0 equiv.) was added, and the mixture was stirred at 40 ºC until complete consumption of **S11** (~5 days). After that, 2 M HCl was added to the solution until pH~1 and the aqueous phase was extracted three times with dichloromethane. The combined organic phases were dried over anhydrous MgSO_4_, filtered, and concentrated under reduced pressure. The product was purified by flash chromatography on silica gel using hexane:EtOAc (10:1) to afford **S19** as a white solid (110 mg, 0.70 mmol, 70% yield). ^1^H- NMR (400 MHz, CD_3_OD) δ, ppm: 2.18 – 2.15 (m, 4H), 1.70 – 1.67 (m, 4H). ^13^C-NMR (101 MHz, CD_3_OD) δ, ppm: 176.2, 61.6, 35.6, 26.4. HRMS (ESI-MS) *m/z* calculated for C_19_H_20_O_4_ [M-Η]^-^ 157.0506, found 157.0504.


**1-((benzyloxy)carbonyl)-4-(*tert*-butyl)cyclohexane-1-carboxylic acid (S21a)**: Substrate **S21a** was prepared according to an analogous procedure for **S13**. Under a N_2_ atmosphere, diisopropylamine (1.9 mL, 20.4 mmol, 2.5 equiv.) was dissolved in anhydrous THF (60 mL). The mixture was cooled to -78 ^o^C and *n*-BuLi (5.4 mL, 2.5 M in hexane, 20.4 mmol, 2.5 equiv.) was added dropwise. After that, *cis*-4-tertbutylcyclohexane carboxylic acid (1.0 g, 5.4 mmol, 1 equiv.) was added. After allowing the mixture to warm to room temperature, it was heated at reflux temperature for 4 h. Then, phenyl chloroformate (0.77 mL, 5.4 mmol, 1 equiv.) was added dropwise. The reaction was then stirred at room temperature overnight, quenched with saturated NH_4_Cl (aq) (5 mL), acidified with 2 M HCl (7 mL), and the product was extracted with Et_2_O (3 x 15 mL). The combined organic phases were dried over anhydrous MgSO_4_, filtered, and concentrated under reduced pressure. The product was purified by flash chromatography on silica gel using hexane:EtOAc (20:1) to afford a mixture of *cis* and *trans* stereoisomers as a white solid (550 mg, 1.70 mmol, 31% yield). Spectroscopical data for single isomer. ^1^H-NMR (400 MHz, CDCl_3_) δ, ppm: 7.36 – 7.27 (m, 5H), 5.15 (s, 2H), 2.45 (d, *J* = 15.5 Hz, 2H), 1.83 – 1.61 (m, 4H), 1.22 – 1.11 (m, 2H), 1.05 – 1.00 (m, 1H), 0.82 (s, 9H). ^13^C-NMR (101 MHz, CDCl_3_) δ, ppm: 176.9, 172.2, 135.7, 128.7, 128.3, 127.7, 67.1, 55.1, 47.1, 32.5, 31.8, 27.5, 23.9. HRMS (ESI-MS) *m/z* calculated for C_19_H_26_O_4_ [M-Η]^-^ 317.1578, found 317.1578.

**4-(*tert*-butyl)cyclohexane-1,1-dicarboxylic acid (S21):** To a round bottom flask **S21a** (550 mg, 1.70 mmol, 1 equiv.) and Pd/C (10% w/w) were added to a MeOH/EtOAc (1:1, 40 mL) mixture. The solvent was first purged with N_2_, and then saturated with H_2_. The mixture was stirred overnight under H_2_ atmosphere (1 atm). The crude mixture was passed through Celite^®^ to remove the Pd/C, and the filtrate was concentrated under reduced pressure. The product was purified by flash chromatography on silica gel using hexane:EtOAc (10:1) to afford **S21** as a white solid (215 mg, 0.94 mmol, 55% yield). ^1^H-NMR (400 MHz, CD_3_OD) δ, ppm: 2.40 (d, *J* = 13.0 Hz, 2H), 1.73 (dd, *J* = 13.4, 3.2 Hz, 2H), 1.61 (td, *J* = 13.3, 3.7 Hz, 2H), 1.19 (qd, *J* = 13.7, 3.6 Hz, 2H), 1.04 (tt, *J* = 12.3, 3.1 Hz, 1H), 0.85 (s, 9H). ^13^C-NMR (101 MHz, CD_3_OD) δ, ppm: 176.3, 174.8, 55.7, 48.54 33.1, 33.0, 27.8, 25.0. HRMS (ESI-) m/z calculated for C_12_H_20_O_4_ [M-Η]^-^ 227.1289, found 227.1282.


 **1-(methoxycarbonyl)-4-methylcyclohexane-1-carboxylic acid (S22a):** Substrate **S22a** was prepared according to an analogous procedure for **S13**. Under a N_2_ atmosphere, diisopropylamine (2.47 mL, 17.6 mmol, 2.5 equiv.) was dissolved in anhydrous THF (80 mL), and the mixture was cooled to -78 °C. *n*-BuLi (7.05 mL, 2.5 M in hexane, 17.6 mmol, 2.5 equiv.) was added dropwise. After that, 4-methylcyclohexanecarboxylic acid (1 g, 7.04 mmol, 1 equiv.) was added. After allowing the mixture to warm to room temperature, it was heated at reflux temperature for 4 h. Then, phenyl chloroformate (0.55 mL, 7.04 mmol, 1.0 equiv.) was added dropwise. The reaction was then stirred at room temperature overnight, quenched with saturated NH_4_Cl (aq) (5 mL), acidified with 2 M HCl (7 mL), and the product was extracted with Et_2_O (3 x 15 mL). The combined organic phases were dried over anhydrous MgSO_4_, filtered, and concentrated under reduced pressure. The product was purified by flash chromatography on silica gel using hexane:EtOAc (20:1) to afford a mixture of *cis* and *trans* stereoisomers along with a small amount of unreacted starting material. The mixture was used directly in the subsequent step without further purification, giving a white solid (400 mg, 2.0 mmol, 29% yield). NMR data for the two isomers (1:1 ratio): ^1^H-NMR (400 MHz, CDCl_3_) δ, ppm: 3.76 (s, 3H), 3.72 (s, 3H), 2.39-2.31 (m, 4H), 1.83 – 1.59 (m, 8H), 1.45 – 1.34 (m, 2H), 1.18 – 0.98 (m, 5H), 0.90-0.87 (m, 7H). ^13^C-NMR (101 MHz, CDCl_3_) δ, ppm: 178.7, 177.2, 172.9, 171.5, 54.8, 54.8, 52.9, 52.7, 31.5, 31.4, 31.3, 31.23, 31.1, 22.2, 22.1. HRMS (ESI-MS) *m/z* calculated for C_10_H16O_4_ [M+Η]^+^ 201.1132, found 201.1122

 **4-methylcyclohexane-1,1-dicarboxylic acid (S22)**: To a solution of **S22a** (400 mg, 2.0 mmol) in MeOH/H_2_O (1:1, 40 mL), NaOH (1.60 g, 40.0 mmol, 20.0 equiv.) was added, and the mixture was stirred at 40 ºC until complete consumption of **S22a** (~5 days). After that, 2 M HCl was added to the solution until pH~1 and the aqueous phase was extracted three times with dichloromethane. The combined organic phases were dried over anhydrous MgSO_4_, filtered, and concentrated under reduced pressure. The product was purified by flash chromatography on silica gel using hexane:EtOAc (10:1) to afford **S22** as a white solid (280 mg, 1.51 mmol, 75% yield). ^1^H-NMR (400 MHz, CD_3_OD) δ, ppm: 2.34 – 2.30 (m, 2H), 1.70 – 1.63 (m, 4H), 1.43-1.35 (m, 1H), 1.15 – 1.05 (m, 2H), 0.89 (d, *J* = 6.6 Hz, 3H). ^13^C-NMR (101 MHz, CD_3_OD) δ, ppm: 176.22, 174.9, 55.6, 32.8, 32.5, 32.3, 22.6. HRMS (ESI-) m/z calculated for C_12_H_20_O_4_ [M-Η]^-^ 185.0819, found 185.081.


 **3,5-dimethylcyclohexane-1-carboxylic acid (S23a):** Substrate **S23a** was prepared according to a slight modification of a reported procedure.^[15]^ 3,5-dimethylbenzoic acid (1.00 g, 6.66 mmol, 1 equiv.) was dissolved in acetic acid (10 mL), after which PtO_2_·H_2_O (100 mg, 10% w/w) was added and the vial was sealed. The vial was placed in a Parr high pressure hydrogenation apparatus and the system was purged with nitrogen for 10 minutes under vigorous stirring. The vessel was then slowly pressurized to 5 bar with hydrogen gas and vented, repeating this process three times. After repressurization to 10 bar, the mixture was stirred overnight. The vessel was then slowly depressurized and opened, and the mixture was filtered through a syringe filter. The acetic acid was evaporated under reduced pressure to afford the **S23a** as a white solid (1.02 mg, 6.53 mmol, 98% yield). ^1^H-NMR (400 MHz, CDCl_3_) δ, ppm 10.80 (brs, 1H) 2.39 (tt, *J* = 12.3, 3.5 Hz, 1H), 2.01 – 1.86 (m, 2H), 1.73 – 1.59 (m, 1H), 1.50 – 1.40 (m, 2H), 1.05 – 0.99 (m, 2H), 0.92 (d, *J* = 6.5 Hz, 6H), 0.58 (q, *J* = 12.0 Hz, 1H). ^13^C-NMR (101 MHz, CDCl_3_) δ, ppm: 181.5, 43.4, 43.1, 36.9, 32.0, 22.5.

 **1-((benzyloxy)carbonyl)-3,5-dimethylcyclohexane-1-carboxylic acid (S23b):** Substrate **S23b** was prepared according to an analogous procedure for **S13**. Under a N_2_ atmosphere, diisopropylamine (1.34 mL, 9.60 mmol, 2.5 equiv.) was dissolved in anhydrous THF (60 mL), and the mixture was cooled to -78 °C. *n*-BuLi (3.84 mL, 2.5 M in hexane, 9.60 mmol, 2.5 equiv.) was added dropwise. After that, **S23a** (600 mg, 3.84 mmol, 1 equiv.) was added. After allowing the mixture to warm to room temperature, it was heated at reflux temperature for 4 h. Then, phenyl chloroformate (0.77 mL, 5.4 mmol, 1.0 equiv.) was added dropwise. The reaction was then stirred at room temperature overnight, quenched with saturated NH_4_Cl (aq) (5 mL), acidified with 2 M HCl (7 mL), and the product was extracted with Et_2_O (3 x 15 mL). The combined organic phases were dried over anhydrous MgSO_4_, filtered, and concentrated under reduced pressure. The product was purified by flash chromatography on silica gel using hexane:EtOAc (25:1) to afford a mixture of *cis* and *trans* stereoisomers as a white solid (470 mg, 1.62 mmol, 42% yield). Spectroscopical data for single isomer. ^1^H-NMR (400 MHz, CDCl_3_) δ, ppm: 7.36 – 7.29 (m, 5H), 5.15 (s, 2H), 2.32 (d, *J* = 13.4 Hz, 2H), 1.66 – 1.57 (m, 3H), 1.29 – 1.23 (m, 2H), 0.91 (d, *J* = 6.3 Hz, 6H), 0.58 (q, *J* = 12.1 Hz, 1H). ^13^C-NMR (101 MHz, CDCl_3_) δ, ppm: 176.8, 172.0, 135.7, 128.7, 128.3, 127.8, 67.2, 56.2, 43.0, 39.0, 29.1, 22.5. HRMS (ESI-MS) *m/z* calculated for C_17_H_22_O_4_ [M-Η]^-^ 289.1445, found 289.1440.

 **3,5-dimethylcyclohexane-1,1-dicarboxylic acid (S23):** To a round bottom **S23b** (470 mg, 1.62 mmol, 1 equiv) and Pd/C (10% w/w) were added to a MeOH/EtOAc (1:1, 40 mL) mixture. The solvent was first purged with N_2_ and then saturated with H_2_. The mixture was stirred overnight under H_2_ atmosphere (1 atm). The crude mixture was passed through Celite^®^ to remove the Pd/C, and the filtrate was concentrated under reduced pressure. The product was purified by flash chromatography on silica gel using hexane:EtOAc (10:1) to afford **S23** as a white solid (260 mg, 1.30 mmol, 80% yield). ^1^H-NMR (400 MHz, CD_3_OD) δ, ppm: 2.29 – 2.24 (m, 2H), 1.67 – 1.52 (m, 3H), 1.15 (dd, *J* = 13.3, 12.1 Hz, 2H), 0.91 (d, *J* = 6.5 Hz, 6H), 0.54 (q, *J* = 11.9 Hz, 1H). ^13^C-NMR (101 MHz, CD_3_OD) δ, ppm: 176.3, 175.2, 57.0, 44.2, 40.5, 30.2, 22.8. HRMS (ESI-MS) *m/z* calculated for C_10_H_16_O_4_ [M-Η]^-^ 199.0976, found 199.0973.


 **cycloheptane-1,1-dicarboxylic acid (S24):** To a solution of **S13** (200 mg, 1.0 mmol) in MeOH/H_2_O (1:1, 40 mL), NaOH (800 mg, 20.0 mmol, 20.0 equiv) was added, and the mixture was stirred at 40 ºC until complete consumption of **S13** (~5 days). After that, 2 M HCl was added to the solution until pH~1 and the aqueous phase was extracted three times with dichloromethane. The combined organic phases were dried over anhydrous MgSO_4_, filtered, and concentrated under reduced pressure. The product was purified by flash chromatography on silica gel using hexane:EtOAc (5:1) to afford **S24** as a white solid (125 mg, 0.67 mmol, 67% yield). ^1^H- NMR (400 MHz, CD_3_OD) δ, ppm: 2.11 – 2.09 (m, 4H), 1.60 – 1.56 (m, 8H). ^13^C-NMR (101 MHz, CD_3_OD) δ, ppm: 176.5, 58.7, 34.95 31.0, 25.0. HRMS (ESI-MS) *m/z* calculated for C_9_H_14_O_4_ [M-Η]^-^ 185.0819, found 185.0824.

**6. Reaction optimization for *δ*-C****-H lactonization of S10**

| **Entry** | **Catalyst** | **Solvent** | **Conv (%)** | **Yield P10 (%)** | **d.r P10** | **Yield δ-P10 (%)** | **d.r δ-P10** | **Ratio P10/ δ-P10** |
| --- | --- | --- | --- | --- | --- | --- | --- | --- |
| 1 | **1^H^** | TFE | 91 | 5 | 1.0 | 8 | 1.0 | 1.6 |
| 2 | **1^TIPS^** | TFE | 70 | 9 | 3.2 | 10 | 2.8 | 1.1 |
| 3 | **2^iPr^** | TFE | 100 | 11 | 2.6 | 37 | 1.8 | 3.4 |
| 4 | **2^iPr^** | HFIP | 100 | 8 | 3.5 | 32 | 1.8 | 4.0 |
| 5 | **2^iPr^** | NFTB | 82 | 8 | 2.2 | 13 | 1.1 | 1.6 |
| 6 | **2^iPr^** | TFE^a^ | 80 | 4 | 2.2 | 12 | 1.8 | 3.0 |
| **Table S1.** *δ*-C−H bond lactonization of 5-phenyl-2-(3-phenylpropyl)-2-((2,2,2-trifluoroethoxy)carbonyl)pentanoic acid (**S10**) under different conditions. General reaction conditions: **S10** (25 mM) and Mn catalyst (2 mol%) were dissolved in the indicated solvent, and H_2_O_2_ (2 equiv., 0.45 M solution in TFE) was independently delivered over 30 min with a syringe pump, at 20 °C, and the reaction was left stirring for additional 30 min. ^a^ In presence of 50 equiv. of AcOH. Conversion, yield, and diastereomeric ratios (d.r.) were determined by GC analysis with biphenyl as internal standard. | | | | | | | | |

**7. Catalytic studies**

7.1. General oxidation protocol

Substrate (25 μmol, 1 molar equivalent) and catalyst (2 mol%) were dissolved in 1 mL of TFE ([substrate] = 25 mM) inside a 12-mL vial equipped with a magnetic stirring bar in an oil bath, unless otherwise indicated. H_2_O_2_ (2 – 4 molar equivalent) solution in TFE (0.45 M, diluted from commercially available H_2_O_2_ water solution 50% w/w, Sigma Aldrich) was delivered over 30 min by syringe pump into the solution under air. (**Note: For safety reasons, the stock of H_2_O_2_ (50% w/w in water) is recommended to be manipulated with precaution due to its corrosive and oxidizing power)**. At the end of the addition, the mixture was stirred for an additional 30 min to ensure complete lactonization. Workup for GC analysis: after quenching the reaction with isopropanol (0.2 mL), biphenyl (12.5 μmol) was added as internal standard, and the mixture was quickly filtered through a short plug of silica gel, which was subsequently rinsed with EtOAc (2 mL). GC analysis of the filtrate provided substrate conversion and product yields relative to the internal standard integration. Isomer ratios were determined by GC.

7.2. General oxidation protocol for product isolation

Catalysis was performed following the general oxidation protocol but in a 0.05-0.56 mmol scale, but without the addition of internal standard. After quenching the reaction with isopropanol, the solvent was removed under reduced pressure, and the resultant crude product was purified by column chromatography. 1D- and 2D-NMR analyses, HRMS, optical rotation and X-ray diffraction analyses were employed for the characterization of the lactones.

**7.3. Characterization of isolated products**

**P1:** Lactone **P1** was isolated with **(*S,S*)**-**2^iPr^** in a 0.25 mmol scale The product was purified by flash chromatography on silica gel using hexane:EtOAc (10:1) to afford **P1** as a pale-yellow oil (23 mg, 0.13 mmol, 53% yield, 70% ee). ^1^H-NMR (400 MHz, CDCl_3_) δ, ppm: 4.33 (dd, *J* = 8.8, 5.4 Hz, 2H), 3.78 (s, 3H), 2.74 (dt, *J* = 13.1, 5.3 Hz, 1H), 2.22 (dt, *J* = 13.2, 8.8 Hz, 1H), 2.17 – 2.00 (m, 1H), 1.89 – 1.80 (m, 1H), 0.96 (t, *J* = 7.5 Hz, 3H). ^13^C-NMR (101 MHz, CDCl_3_) δ, ppm: 174.9, 170.2, 66.24 54.8, 53.2, 31.3, 27.4, 9.23 ${[\alpha]}_{D}^{20}$= +2.22 (c = 0.256, CHCl_3_). HRMS (ESI-MS) *m/z* calculated for C_8_H_12_O_4_ [M+Η]^+^ 173.0808, found 173.0810.

**P2:** Lactone **P2** was isolated with **(*S,S*)-2^iPr^** in a 0.527 mmol scale. The product was purified by flash chromatography c silica gel using hexane:EtOAc (20:1) to afford **P2** as a pale-yellow oil (60 mg, 0.32 mmol, 61% yield, 55% ee). Spectroscopic data matched with previous reported ones.^[16]^ ^1^H-NMR (400 MHz, CDCl_3_) δ, ppm: 4.34 – 4.31 (m, 2H), 4.23 (dtt, *J* = 10.8, 7.4, 3.7 Hz, 2H), 2.75 – 2.69 (m, 1H), 2.26 – 2.18 (m, 1H), 2.16 – 2.06 (m, 1H), 1.89 – 1.79 (m, 1H), 1.28 (t, *J* = 7.1 Hz, 3H), 0.96 (t, *J* = 7.5 Hz, 3H). ^13^C-NMR (101 MHz, CDCl_3_) δ, ppm: 175.0, 169.7, 66.2, 62.3, 54.8, 31.4, 27.3, 14.2, 9.2.
${[\alpha]}_{D}^{20}$= +12.96 (c = 0.417, CHCl_3_). HRMS (ESI-MS) *m/z* calculated for C_9_H_14_O_4_ [M+H]^+^ 187.0965, found 187.0969.

**P3:** Lactone **P3** was isolated with **(*S,S*)-2^iPr^** in a 0.372 mmol scale. The product was purified by flash chromatography on silica gel using hexane:EtOAc (15:1) to afford **P3** as a colorless oil (60 mg, 0.25 mmol, 67% yield, 56% ee). ^1^H-NMR (400 MHz, CDCl_3_) δ, ppm: 4.57 (qd, *J* = 8.3, 4.5 Hz, 2H), 4.37 (dd, *J* = 8.5, 5.7 Hz, 2H), 2.75 (dt, *J* = 13.3, 5.7 Hz, 1H), 2.30 (dt, *J* = 13.3, 8.5 Hz, 1H), 2.22 – 2.12 (m, 1H), 1.90 (dq, *J* = 14.9, 7.5 Hz, 1H), 0.99 (t, *J* = 7.5 Hz, 3H). ^13^C-NMR (101 MHz, CDCl_3_) δ, ppm: 173.9, 168.4, 122.7 (q, *J* = 277.4 Hz), 66.2, 61.2 (q, *J* = 37.0 Hz), 54.7, 31.2, 27.2, 9.0. ^19^F-NMR (377 MHz, CDCl_3_) δ, ppm: -74.7. ${[\alpha]}_{D}^{20}$= +2.15 (c = 0.288, CHCl_3_). HRMS (ESI-MS) *m/z* calculated for C_9_H_11_F_3_O_4_ [M+H]^+^ 241.0682, found 241.0684.

**P4:** Lactone **P4** was isolated with **(*S,S*)-2^iPr^** in a 0.50 mmol scale. The product was purified by flash chromatography on silica gel using hexane:EtOAc (20:1) to afford **P4** as a colorless oil as a mixture of diastereomers (84 mg, 0.43 mmol, 84% yield, d.r.= 6.0, >99.9% ee for the major and 5% for the minor diastereomer). *Spectroscopical data for the minor diastereomer:* ^1^H-NMR (400 MHz, CDCl_3_) δ, ppm: 4.65 – 4.56 (m, 1H), 3.75 (s, 3H), 2.81 (dd, *J* = 13.0, 5.6 Hz, 1H), 2.09 (ddd, *J* = 13.7, 12.1, 4.7 Hz, 1H), 1.74 – 1.65 (m, 2H), 1.30 (t, *J* = 6.3 Hz, 3H), 1.39 – 1.30 (m, 2H), 0.95 (t, *J* = 7.3 Hz, 3H). ^13^C-NMR (101 MHz, CDCl_3_) δ, ppm: 174.3, 170.2, 75.2, 56.8, 53.2, 39.9, 36.9, 20.9, 18.4, 14.2. *Spectroscopical data for the major diastereomer:* ^1^H-NMR (400 MHz, CDCl_3_) δ, ppm: 4.61 (ddt, *J* = 13.6, 7.3, 6.3 Hz, 1H), 3.78 (s, 3H), 2.46 (dd, *J* = 13.3, 7.5 Hz, 1H), 2.36 (dd, *J* = 13.4, 7.1 Hz, 1H), 1.97 (ddd, *J* = 13.7, 11.9, 5.1 Hz, 1H), 1.82 (ddd, *J* = 13.7, 11.6, 5.0 Hz, 1H), 1.42 (d, *J* = 6.3 Hz, 3H), 1.40 – 1.31 (m, 2H), 0.95 (t, *J* = 7.3 Hz, 3H). ^13^C-NMR (101 MHz, CDCl_3_) δ, ppm: 174.8, 170.9, 74.6, 55.8, 53.2, 38.7, 36.5, 21.4, 18.3, 14.23. ${[\alpha]}_{D}^{20}$= -1.91 (c = 0.346, CHCl_3_). HRMS (ESI-MS) *m/z* calculated for C_10_H_16_O_4_ [M+H]^+^ 201.1122, found 201.1121.

**P5:** Lactone **P5** was isolated with **(*R,R*)-2^iPr^** in a 0.1 mmol scale. The product was purified by flash chromatography on silica gel using hexane:EtOAc (50:1) to afford **P5** as a white solid (22 mg, 0.07 mmol, d.r.= 29.4, 70% yield, >99.9% for the major diastereomer and 40% for the minor). ^1^H-NMR (400 MHz, CDCl_3_) δ, ppm: 7.43 – 7.35 (m, 5H), 7.33 – 7.29 (m, 2H), 7.24 – 7.21 (m, 3H), 5.52 (t, *J* = 7.7 Hz, 1H), 3.72 (s, 3H), 2.91 – 2.84 (m, 1H), 2.84 – 2.76 (m, 1H), 2.76 – 2.68 (m, 2H), 2.41 – 2.25 (m, 2H). ^13^C-NMR (101 MHz, CDCl_3_) δ, ppm: 174.2, 170.3, 140.6, 138.8, 128.9, 128.8, 128.7, 128.5, 126.5, 125.6, 78.7, 55.4, 53.2, 40.5, 35.8, 31.3. ${[\alpha]}_{D}^{20}$= +13.8 (c = 0.257, CHCl_3_). HRMS (ESI-MS) m/z calculated for C_20_H_20_O_4_ [M+H]^+^ 325.1434, found 325.1421.

**P6:** Lactone **P6** was isolated with **(*R,R*)-2^iPr^** in a 0.378 mmol scale. The product was purified by flash chromatography on silica gel using hexane:EtOAc (20:1) to afford lactone **P6** as a pale-yellow oil (47 mg, 0.21 mmol, 55% yield, 87% ee). ^1^H-NMR (400 MHz, CDCl_3_) δ, ppm: 3.76 (s, 3H), 2.89 (d, *J* = 13.4 Hz, 1H), 2.24 – 2.20 (m, 1H), 2.03 (d, *J* = 13.4 Hz, 1H), 1.70 – 1.59 (m, 2H), 1.45 (s, 3H), 1.36 (s, 3H), 0.89 (t, *J* = 6.0 Hz, 6H). ^13^C-NMR (101 MHz, CDCl_3_) δ, ppm:174.3, 171.2, 82.2, 56.6, 53.3, 45.3, 43.6, 30.1, 28.2, 25.7, 23.5, 23.0. ${[\alpha]}_{D}^{20}$= +7.64 (c = 0.284, CHCl_3_). HRMS (ESI-MS) *m/z* calculated for C_12_H_20_O_4_ [M+H]^+^ 229.1434, found 229.1428.

**P7:** Lactone **P7** was isolated using **(*R,R*)-2^iPr^** in a 0.25 mmol scale. The product was purified by flash chromatography on silica gel using hexane:EtOAc (50:1) to afford **P7** as a colorless oil as a mixture of diastereomers (45 mg, 0.20 mmol, 79% yield, dr.r.= 8.0, 95% ee). *Spectroscopical data for the minor diastereomer:* ^1^H-NMR (400 MHz, CDCl_3_) δ, ppm: 4.46 – 4.41 (m, 1H), 3.76 (s, 3H), 2.77 (dd, *J* = 13.0, 5.7 Hz, 1H), 2.12 (ddd, *J* = 13.8, 12.2, 4.4 Hz, 1H). 1.88 – 1.80 (m, 1H), 1.68 – 1.61 (m, 1H), 1.37 – 1.27 (m, 4H), 1.01 (t, *J* = 7.5 Hz, 3H), 0.89 (t, *J* = 6.8 Hz, 3H). ^13^C-NMR (101 MHz, CDCl_3_) δ, ppm: 174.4, 170.4, 80.1, 56.4, 55.2, 37.7, 28.5, 27.1, 22.9, 9.6. *Spectroscopical data for the major diastereomer:* ^1^H-NMR (400 MHz, CDCl_3_) δ, ppm: 4.35 (quin, *J* = 7.6 Hz, 1H), 3.76 (s, 3H), 2.47 (dd, *J* = 13.3, 7.8 Hz, 1H), 2.30 (dd, *J* = 13.3, 7.1 Hz, 1H), 2.02 – 1.95 (m, 1H), 1.90 – 1.83 (m, 1H), 1.80 – 1.74 (m, 1H), 1.68 – 1.62 (m, 1H), 1.36 – 1.26 (m, 4H), 0.99 (t, *J* = 7.5 Hz, 3H), 0.89 (t, *J* = 6.8 Hz, 3H). ^13^C-NMR (101 MHz, CDCl_3_) δ, ppm: 174.8, 170.9, 79.5, 55.5, 53.1, 36.7, 34.3, 28.8, 27.0, 22.9, 13.9, 9.6. ${[\alpha]}_{D}^{20}$= +6.20 (c = 0.275, CHCl_3_). HRMS (ESI-MS) *m/z* calculated for C_12_H_20_O_4_ [M+H]^+^ 229.1434, found 229.1427.

**δ-P7:** Lactone **δ-P7** was isolated using **(*R,R*)-2^iPr^** in a 0.25 mmol scale. The product was purified by flash chromatography on silica gel using hexane:EtOAc (50:1) to afford **δ-P7** as a colorless oil (4 mg, 0.018 mmol, 7% yield, dr.r. = 1.0). ^1^H-NMR (400 MHz, CDCl_3_) δ, ppm: 4.37 – 4.29 (m, 1H), 3.76 (s, 3H), 2.49 (dt, *J* = 14.0, 8.0 Hz, 1H), 2.01 – 1.93 (m, 2H), 1.86 – 1.72 (m, 2H), 1.66 – 1.53 (m, 2H), 1.34 (d, *J* = 6.2 Hz, 3H), 1.33 – 1.28 (m, 3H), 0.90 (t, *J* = 7.0 Hz, 3H). ^13^C-NMR (101 MHz, CDCl_3_) δ, ppm: 172.1, 171.1, 74.8, 53.1, 36.4, 29.8, 27.9, 26.9, 26.6, 23.0, 21.5, 14.0. HRMS (ESI-MS) *m/z* calculated for C_12_H_20_O_4_ [M+H]^+^ 229.1434, found 229.1427.

**P8:** Lactone **P8** was isolated using **(*R,R*)-2^iPr^** in a 0.375 mmol scale. The product was purified by flash chromatography on silica gel using hexane:EtOAc (50:1) to afford **P8** as a colorless oil as a mixture of diastereomers (53 mg, 0.23 mmol, 55 % yield, dr.r.= 9.1, 92% ee). *Spectroscopical data for the minor diastereomer:* ^1^H-NMR (400 MHz, CDCl_3_) δ, ppm: 4.17 (ddd, *J* = 10.4, 7.7, 5.7 Hz, 1H), 3.76 (s, 3H), 2.70 (dd, *J* = 13.0, 5.7 Hz, 1H), 2.12 (ddd, *J* = 13.7, 12.4, 4.8 Hz, 1H), 1.89 – 1.80 (m, 1H), 1.03 (d, *J* = 6.7 Hz, 3H), 0.93 (d, *J* = 6.7 Hz, 3H), 0.88 (d, *J* = 6.6 Hz, 6H). ^13^C-NMR (101 MHz, CDCl_3_) δ, ppm: 174.46, 170.34, 83.65, 56.50, 53.23, 35.95, 33.85, 33.10, 32.78, 28.30, 22.57, 22.42, 18.78, 17.34. *Spectroscopical data for the major diastereomer:* ^1^H-NMR (400 MHz, CDCl_3_) δ, ppm: 4.11 (q, *J* = 7.8 Hz, 1H), 3.77 (s, 3H), 2.53 (dd, *J* = 13.4, 8.7 Hz, 1H), 2.22 (dd, *J* = 13.4, 7.0 Hz, 1H), 2.08 – 1.93 (m, 1H), 1.91 – 1.78 (m, 2H), 1.55 (dt, *J* = 13.3, 6.6 Hz, 1H), 1.32 – 1.14 (m, 2H), 1.03 (d, *J* = 6.7 Hz, 3H), 0.93 (d, *J* = 6.7 Hz, 3H), 0.89 (d, *J* = 6.6 Hz, 6H). ^13^C-NMR (101 MHz, CDCl_3_) δ, ppm: 174.9, 170.9, 83.2, 55.7, 53.1, 35.1, 33.7, 33.3, 32.4, 28.4, 22.6, 22.4, 18.7, 17.5. ${[\alpha]}_{D}^{20}$= +1.78 (c = 0.486, CHCl_3_). HRMS (ESI-MS) *m/z* calculated for C_14_H_24_O_4_ [M+H]^+^ 257.1747, found 257.1738.

**δ-P8:** Lactone **δ-P8** was isolated with **(*R,R*)-2^iPr^** in a 0.375 mmol scale. The product was purified by flash chromatography on silica gel using hexane:EtOAc (50:1) to afford lactone **δ-P8** as a colorless oil (13 mg, 0.051 mmol, 14% yield). ^1^H-NMR (400 MHz, CDCl_3_) δ, ppm: 3.75 (s, 3H), 2.23 – 2.16 (m, 1H), 2.02 – 1.90 (m, 3H), 1.87 – 1.73 (m, 2H), 1.53 (hept, *J* = 6.6 Hz, 1H), 1.43 (s, 3H), 1.39 (s, 3H), 1.22 – 1.13 (m, 2H), 0.89 (d, *J* = 6.6 Hz, 6H). ^13^C-NMR (101 MHz, CDCl_3_) δ, ppm:172.5, 170.2, 83.3, 53.6, 52.9, 34.2, 33.6, 32.0, 29.9, 28.5, 28.3, 26.2, 22.6, 22.5. HRMS (ESI-MS) *m/z* calculated for C_14_H_24_O_4_ [M+H]^+^ 257.1747, found 257.1741.

**P9:** Lactone **P9** was isolated with **(*R,R*)-2^iPr^** in a 0.25 mmol scale. The product was purified by flash chromatography on silica gel using hexane:EtOAc (50:1) to afford **P9** as a colorless oil as a mixture of diastereomers (15 mg, 0.04 mmol, d.r.=5.6, 16% yield, 99.9% ee for the major diastereomer and 22% for the minor). *Spectroscopical data for the minor diastereomer; please note that, due to overlap with signals from the major isomer, only the following resonances could be unambiguously assigned:* ^1^H-NMR (400 MHz, CDCl_3_) δ, ppm: 4.73 (dd, *J* = 10.1, 6.1 Hz, 1H), 3.04 (dd, *J* = 14.1, 6.5 Hz, 2H), 2.93 (dd, *J* = 14.1, 5.7 Hz, 1H). *Spectroscopical data for the major diastereomer:* ^1^H-NMR (400 MHz, CDCl_3_) δ, ppm: 7.34 – 7.31 (m, 2H), 7.28 – 7.22 (m, 5H), 7.18 – 7.13 (m, 3H), 4.61 (quint, *J* = 7.1 Hz, 1H), 3.77 (s, 3H), 3.13 (dd, *J* = 13.8, 6.5 Hz, 1H), 2.87 (dd, *J* = 13.8, 7.0 Hz, 1H), 2.64 – 2.56 (m, 3H), 2.19 (dd, *J* = 13.5, 7.2 Hz, 1H), 2.03 (ddd, *J* = 13.7, 12.1, 4.8 Hz, 1H), 1.90 – 1.83 (m, 1H), 1.69 – 1.60 (m, 2H). ^13^C-NMR (101 MHz, CDCl_3_) δ, ppm: 174.4, 170.7, 141.3, 135.9, 129.5, 128.8, 128.5, 128.5, 127.2, 126.2, 78.5, 55.3, 53.3, 41.6, 36.3, 35.8, 34.1, 26.7. HRMS (ESI-MS) *m/z* calculated for C_22_H_24_O_4_ [M+H]^+^ 353.1747, found 353.1754.

**δ-P9:** Lactone **δ-P9** was isolated with **(*R,R*)-2^iPr^** in a 0.25 mmol scale. The product was purified by flash chromatography on silica gel using hexane:EtOAc (50:1) to afford **δ-P9** as a colorless oil as a mixture of diastereomers (30 mg, 0.08 mmol, d.r.=1.7, 36% yield, 98% ee for the major diastereomer and n.d. for the minor). *Spectroscopical data for the major diastereomer:* ^1^H-NMR (400 MHz, CDCl_3_) δ, ppm: 7.37 – 7.27 (m, 7H), 7.21 – 7.19 (m, 3H), 5.32 – 5.28 (m, 1H), 3.79 (s, 3H), 2.73 – 2.64 (m, 2H), 2.31 – 2.28 (m, 1H), 2.17 (ddd, *J* = 13.7, 12.3, 4.4 Hz, 1H), 2.08 – 1.99 (m, 3H), 1.83 – 1.79 (m, 1H), 1.73 – 1.62 (m, 2H). ^13^C-NMR (101 MHz, CDCl_3_) δ, ppm: 172.3, 169.6, 141.8, 139.7, 128.8, 128.6, 128.5, 128.5, 126.1, 125.9, 83.4, 54.4, 53.1, 36.1, 36.1, 29.5, 29.3, 26.8. HRMS (ESI-MS) *m/z* calculated for C_22_H_24_O_4_ [M+H]^+^ 353.1747, found 353.1745.

 **P10:** Lactone **P10** was isolated with **(*R,R*)-2^iPr^** in a 0.260 mmol scale. The product was purified by flash chromatography on silica gel using hexane:EtOAc (50:1) to afford **P10** as a colorless oil as a mixture of diastereomers. Due to low yield and the formation of 4 lactones (2 *γ*-lactones and 2 *δ*-lactones), **P10** could not be isolated in a pure form. However, its assignation as a *γ*-lactone was based on ^1^H NMR, which shows that the peaks in the characteristic 4.70 – 4.65 ppm region correspond to *γ*-lactones. **P10** was obtained in 11% yield (determined by GC) with a d.r.=2.6. >99.9% ee for the major diastereomer and 22% for the minor). HRMS (ESI-MS) *m/z* calculated for C_23_H_23_F_3_O_4_ [M+H]^+^ 421.1621, found 421.1615.

**δ-P10:** Lactone **δ-P10** was isolated with **(*R,R*)-2^iPr^** in a 0.260 mmol scale. The crude product was purified by flash chromatography on silica gel using hexane:EtOAc (50:1) to afford **δ-P10** as a colorless oil as a mixture of diastereomers (43 mg, 0.10 mmol, d.r.=1.8, 37% yield, 97% ee for the major diastereomer and 93% for the minor). *Spectroscopical data for the major diastereomer:* ^1^H-NMR (400 MHz, CDCl_3_) δ, ppm: 7.40 – 7.28 (m, 7H), 7.23 – 7.19 (m, 3H), 5.34 – 5.30 (m, 1H), 4.69 (dq, *J* = 12.7, 8.3 Hz, 1H), 4.48 (dq, *J* = 12.7, 8.3 Hz, 1H), 2.74 – 2.64 (m, 2H), 2.32 – 2.26 (m, 1H), 2.24 – 2.17 (m, 1H), 2.13 – 2.00 (m, 4H), 1.90 – 1.80 (m, 1H), 1.75 – 1.66 (m, 1H). ^13^C-NMR (101 MHz, CDCl_3_) δ, ppm: 170.3, 168.6, 141.4, 139.2, 128.7, 128.6, 128.5, 128.4, 126.1, 125.8, 122.7 (q, *J* = 277.5 Hz) , 83.4, 61.0 (q, *J* = 36.8 Hz), 36.0, 35.9, 29.2, 28.9, 26.6. ^19^F-NMR (377 MHz, CDCl_3_) δ, ppm: -74.5. ${[\alpha]}_{D}^{20}$= +5.14 (c = 0.224, CHCl_3_). HRMS (ESI-MS) *m/z* calculated for C_23_H_23_F_3_O_4_ [M+H]^+^ 421.1621, found 421.1615.

**P11:** Lactone **P11** was isolated with **(*R,R*)-2^iPr^** in a 0.5 mmol scale. The product was purified by flash chromatography on silica gel using hexane:EtOAc (15:1) to afford **P11** as a white solid (28 mg, 0.17 mmol, 33% yield, 66% ee). ^1^H-NMR (400 MHz, CDCl_3_) δ, ppm: 4.92 (br, 1H), 3.81 (s, 3H), 2.54 – 2.50 (m, 1H), 2.25 – 2.16 (m, 1H), 2.07 – 1.98 (m, 3H), 1.95 (d, *J* = 10.5 Hz, 1H). ^13^C-NMR (101 MHz, CDCl_3_) δ, ppm: 173.6, 168.7, 80.0, 56.8, 52.8, 43.3, 29.3, 26.0. ${[\alpha]}_{D}^{20}$= -45.02 (c = 0.357, CHCl_3_). HRMS (ESI-MS) *m/z* calculated for C_8_H_10_O_4_ [M+H]^+^ 171.0652, found 171.0653.

**P12:** Lactone **P12** was isolated with **(*R,R*)-2^iPr^** in a 0.56 mmol scale. The product was purified by flash chromatography on silica gel using hexane:EtOAc (10:1) to afford **P12** as a white solid (68 mg, 0.37 mmol, 67% yield, >99.9% ee). ^1^H-NMR (400 MHz, CDCl_3_) δ, ppm: 4.90 – 4.88 (m, 1H), 3.78 (s, 3H), 2.91 (ddt, *J* = 11.6, 6.3, 2.3 Hz, 1H), 2.35 – 2.25 (m, 1H), 2.09 – 1.96 (m, 1H), 1.91 (d, *J* = 11.6 Hz, 2H), 1.80 – 1.71 (m, 2H), 1.57 – 1.49 (m, 1H). ^13^C-NMR (101 MHz, CDCl_3_) δ, ppm: 174.2, 170.1, 76.7, 53.0, 52.9, 41.3, 29.2, 27.4, 18.2. ${[\alpha]}_{D}^{20}$= -49.25 (c = 0.298, CHCl_3_). HRMS (ESI-MS) *m/z* calculated for C_9_H_12_O_4_ [M+H]^+^ 185.0808, found 185.0814.

**P13:** Lactone **P13** was isolated with **(R,R)-2^iPr^** in a 0.5 mmol scale. The product was purified by flash chromatography on silica gel using hexane:EtOAc (10:1) to afford **P13** as a white solid (67 mg, 0.34 mmol, 68% yield, 99% ee). ^1^H-NMR (400 MHz, CDCl_3_) δ, ppm: 4.95 (dt, *J* = 8.6, 3.0 Hz, 1H), 3.78 (s, 3H), 2.95 (dd, *J* = 13.0, 8.6 Hz, 1H), 2.34 – 2.27 (m, 1H), 2.24 (d, *J* = 13.0 Hz, 1H), 2.08 – 2.01 (m, 2H), 1.76 – 1.60 (m, 5H). ^13^C-NMR (101 MHz, CDCl_3_) δ, ppm: 176.5, 171.2, 78.1, 54.4, 53.2, 35.1, 34.2, 33.7, 23.9, 22.8. ${[\alpha]}_{D}^{20}$= -26.27 (c = 0.275, CHCl_3_). HRMS (ESI-MS) *m/z* calculated for C_10_H_14_O_4_ [M+H]^+^ 199.0965, found 199.0961.

**P14:** Lactone **P14** was isolated with **(R,R)-2^iPr^** in a 0.355 mmol scale. The product was purified by flash chromatography on silica gel using hexane:EtOAc (25:1) to afford **P14** as a yellow oil (31 mg, 0.15 mmol, 41% yield, 98% ee). ^1^H-NMR (400 MHz, CDCl_3_) δ, ppm: 4.90 (br, 1H), 3.73 (s, 3H), 3.03 (t, *J* = 12.3 Hz, 1H), 2.43 (d, *J* = 14.4 Hz, 1H), 2.25 – 2.18 (m, 2H), 2.13 – 2.06 (m, 1H), 1.98 – 1.79 (m, 3H), 1.61 – 1.47 (m, 2H), 1.40 – 1.22 (m, 2H). ^13^C- NMR (101 MHz, CDCl_3_) δ, ppm: 175.5, 172.1, 75.9, 54.1, 53.2, 37.5, 35.9, 34.5, 28.0, 25.4, 25.2. ${[\alpha]}_{D}^{20}$= -29.29 (c = 0.420, CHCl_3_). HRMS (ESI-MS) *m/z* calculated for C_11_H_16_O_4_ [M+H]^+^ 213.1121, found 213.1117.

**P15:** Lactone **P15** was isolated with **(*R,R*)-2^iPr^** in a 0.478 mmol scale. The product was purified by flash chromatography on silica gel using hexane:EtOAc (50:1) to afford **P15** along with the unreacted **S15** substrate as a white solid (29% yield, *rac*). ^1^H-NMR (400 MHz, CDCl_3_) δ, ppm: 4.44 – 4.33 (m, 2H), 2.80 – 2.74 (m, 1H), 2.28 (dt, *J* = 13.3, 8.3 Hz, 1H), 2.12 (dq, *J* = 14.7, 7.5 Hz, 1H), 1.87 (dt, *J* = 14.5, 7.3 Hz, 1H), 1.01 (t, *J* = 7.4 Hz, 3H). ^13^C-NMR (101 MHz, CDCl_3_) δ, ppm: 175.5, 174.6, 66.6, 54.9, 31.0, 27.4, 9.2. HRMS (ESI-MS) *m/z* calculated for C_7_H_10_O_4_ [M+Na]^+^ 181.0471, found 181.0479.

 ***bis*-P16^syn-sym^:** Lactone ***bis*-P16^syn-sym^** was isolated with **(*R,R*)-2^iPr^** in a 0.375 mmol scale. The product was purified by flash chromatography on silica gel using hexane:EtOAc (40:1) to afford ***bis*-P16^syn-sym^** as white solid (14 mg, 0.075 mmol, 20% yield, >99.9 % ee). Spectroscopic data matched with previous reported ones.^[17]^ ^1^H-NMR (400 MHz, CDCl_3_) δ, ppm: 5.04 (m, 2H), 2.83 (dd, *J* = 13.1, 6.2 Hz, 2H), 1.89 (dd, *J* = 13.1, 9.4 Hz, 2H), 1.46 (d, *J* = 6.2 Hz, 6H). ^13^C-NMR (101 MHz, CDCl_3_) δ, ppm: 173.6, 76.0, 40.6, 21.0. ${[\alpha]}_{D}^{20}$= -47.6 (c = 0.208, CHCl_3_). HRMS (ESI-MS) *m/z* calculated for C_9_H_12_O_4_ [M+H]^+^ 185.0808, found 185.0810.

 ***bis*-P16^unsym^:** Lactone ***bis*-P16^unsym^** was isolated with **(*R,R*)-2^iPr^** in a 0.375 mmol scale. The product was purified by flash chromatography on silica gel using hexane:EtOAc (40:1) to afford ***bis*-P16^unsym^** as white solid (20% yield, 14 % ee). Spectroscopic data matched with previous reported ones.^[17]^ ^1^H-NMR (400 MHz, CDCl_3_) δ, ppm: 5.05 – 4.93 (m, 1H), 4.74 – 4.66 (m, 1H), 2.81 (dd, *J* = 12.9, 5.7 Hz, 1H), 2.60 (dd, *J* = 13.4, 8.1 Hz, 1H), 2.33 (dd, *J* = 13.4, 6.8 Hz, 1H), 1.90 (dd, *J* = 12.9, 9.9 Hz, 1H), 1.53 (d, *J* = 6.2 Hz, 3H), 1.46 (d, *J* = 6.2 Hz, 3H). ^13^C-NMR (101 MHz, CDCl_3_) δ, ppm: 173.9, 173.7, 76.1, 75.2, 54.1, 41.8, 39.7, 21.2, 20.7.

 ***bis*-P16^anti-sym^:** Lactone ***bis*-P16^anti-sym^** was isolated with **(*R,R*)-2^iPr^** in a 0.375 mmol scale. The product was purified by flash chromatography on silica gel using hexane:EtOAc (40:1) to afford ***bis*-P16^anti-sym^** as white solid in 20% yield. ee could not be precisely determined. Spectroscopic data matched with previous reported ones.^[17]^ ^1^H-NMR (400 MHz, CDCl_3_) δ, ppm: 4.75 – 4.62 (m, 2H), 2.47 – 2.39 (m, 4H), 1.53 (d, *J* = 6.3 Hz, 6H). ^13^C-NMR (101 MHz, CDCl_3_) δ, ppm: 174.5, 75.6, 53.4, 40.8, 21.0.

**P17:** Lactone **P17** was isolated with **(*S,S*)-2^iPr^** in a 0.250 mmol scale. The product was purified by flash chromatography on silica gel using hexane:EtOAc (50:1) as white solid as a mixture of diastereomers (37 mg, 0.119 mmol, 48% yield, dr.r.= 3.3, 99.9% ee for the major and 71% for the minor diastereomer). *Spectroscopical data for the minor diastereomer:* ^1^H-NMR (400 MHz, CDCl_3_) δ, ppm: 7.43 – 7.34 (m, 5H), 7.35 – 7.28 (m, 2H), 7.25 – 7.18 (m, 3H), 5.64 (dd, *J* = 10.1, 6.1 Hz, 1H), 3.18 (dd, *J* = 13.4, 6.1 Hz, 1H), 2.69 – 2.52 (m, 4H), 2.43 – 2.31 (m, 2H). *Spectroscopical data for the major diastereomer:* ^1^H-NMR (400 MHz, CDCl_3_) δ, ppm: 7.43 – 7.36 (m, 5H), 7.33 – 7.30 (m, 2H), 7.26 – 7.22 (m, 3H), 5.53 (dd, *J* = 9.2, 6.9 Hz, 1H), 2.91 – 2.73 (m, 4H), 2.42 – 2.27 (m, 2H). ^13^C-NMR (101 MHz, CDCl_3_) δ, ppm: 174.9, 173.8, 140.2, 138.1, 129.2, 129.1, 128.8, 128.5, 126.7, 125.9, 79.3, 55.7, 40.2, 36.0, 31.3. ${[\alpha]}_{D}^{20}$= -20.3 (c = 0.357, CHCl_3_). HRMS (ESI-MS) m/z calculated for C_19_H_18_O_4_ [M+Na]^+^ 333.1097, found 333.1091.

 ***bis*-P17:** Bis-lactone ***bis*-P17** was isolated with **(*S,S*)-2^iPr^** in a 0.054 mmol scale. The product was purified by flash chromatography on silica gel using hexane:EtOAc (50:1) as a white solid as a mixture of three diastereomers (35% total yield, dr.r.= 1:2:10, 99.9% ee for the major). *Spectroscopical data for the major diastereomer* ***bis*-P17^anti-sym^:** ^1^H-NMR (400 MHz, CDCl_3_) δ, ppm: 7.44 – 7.39 (m, 10H), 5.52 (dd, *J* = 9.6, 6.6 Hz, 2H), 2.90 – 2.78 (m, 4H). ^13^C-NMR (101 MHz, CDCl_3_) δ, ppm: 174.1, 137.5, 129.4, 129.2, 126.1, 80.0, 54.0, 41.8. HRMS (ESI-MS) m/z calculated for C_19_H_16_O_4_ [M+H]^+^ 309.1121, found 309.1133.

***bis*-P18:** Bis-lactone ***bis*-P18** was isolated with **(*R,R*)-2^iPr^** in a 0.250 mmol scale. The product was purified by flash chromatography on silica gel using hexane:EtOAc (20:1) to afford ***bis*-P18** as a white solid (37 mg, 0.075 mmol, 70% yield, 35% ee). Spectroscopic data matched with previous reported ones.^[18]^ ^1^H-NMR (400 MHz, CDCl_3_) δ, ppm: 2.83 (d, *J* = 13.4 Hz, 1H), 2.16 (d, *J* = 13.4 Hz, 1H), 1.61 (s, 6H), 1.47 (s, 6H). ^13^C-NMR (101 MHz, CDCl_3_) δ, ppm: 174.2, 83.6, 48.0, 29.6, 28.6. ${[\alpha]}_{D}^{20}$= +45.6 (c = 0.212, CHCl_3_). HRMS (ESI-MS) *m/z* calculated for C11H_16_O_4_ [M+H]^+^ 213.1121, found 213.1125.

**P20:** Lactone **P20** was isolated with **(*R,R*)-2^iPr^** in a 0.256 mmol scale. The product was purified by flash chromatography on silica gel using hexane:EtOAc (50:1) to afford **P20** as white solid (22 mg, 0.13 mmol, 51% yield, 98% ee). ^1^H-NMR (400 MHz, CDCl_3_) δ, ppm: 8.55 (br, 1H), 4.93 (t, *J* = 5.2 Hz, 1H), 2.90 – 2.84 (m, 1H), 2.29 – 2.24 (m, 1H), 2.07 – 1.99 (m, 2H), 1.95 – 1.91 (m, 1H), 1.85 – 1.71 (m, 2H), 1.57 (ddd, *J* = 13.6, 11.5, 6.2 Hz, 1H). ^13^C-NMR (101 MHz, CDCl_3_) δ, ppm: 175.0, 173.9, 77.4, 52.7, 40.7, 29.6, 27.2, 18.2. ${[\alpha]}_{D}^{20}$= +55.0 (c = 0.315, CHCl_3_). HRMS (ESI-MS) *m/z* calculated for C_8_H_10_O_4_ [M+H]^+^ 171.0652, found 171.0645.

**P21:** Yield of **P21** was determined by NMR using 1,3,5-trimethoxybenzene as internal standard (0.5 equiv.). The characterization of the product and the determination of ee was performed after derivatization (See section **SFC and GC traces**).

 **P22:** Yield of **P22** determined by NMR using 1,3,5-trimethoxybenzene as internal standard (0.5 equiv.). The characterization of the product and the determination of ee was performed after derivatization (See section **SFC and GC traces**).

 **P23:** Yield of **P23** determined by NMR using 1,3,5-trimethoxybenzene as internal standard (0.5 equiv.). The characterization of the product and the determination of ee was performed after derivatization (See section **SFC and GC traces**).

**P24:** Lactone **P24** was isolated with **(*R,R*)-2^iPr^** in a 0.250 mmol scale. The product was purified by flash chromatography on silica gel using hexane:EtOAc (50:1) to afford **P24** as a white solid (34 mg, 0.13 mmol, 72% yield, 88% ee). ^1^H-NMR (400 MHz, CDCl_3_) δ, ppm 9.28 (br, 1H), 4.99 – 4.96 (m, 1H), 2.93 (dd, *J* = 13.3, 8.6 Hz, 1H), 2.34 – 2.26 (m, 2H), 2.08 – 2.00 (m, 2H), 1.79 – 1.61 (m, 5H). ^13^C-NMR (101 MHz, CDCl_3_) δ, ppm: 177.6, 174.6, 78.8, 54.2, 35.3, 34.4, 33.4, 24.0, 22.7. ${[\alpha]}_{D}^{20}$= +21.9 (c = 0.253, CHCl_3_). HRMS (ESI-MS) *m/z* calculated for C_9_H_12_O_4_ [M-H]^-^ 183.0663, found 183.0661.

**7.4. Product elaboration**

**6-oxabicyclo[3.2.1]octan-7-one (P25):** It was prepared according to a slight modification of a reported procedure.^[19]^ Lactone **P20** (25 mg, 1 equiv.) and *p*-toluenesulfonic acid monohydrate (56 mg, 2 equiv.) were dissolved in DMSO (3 mL) and heated at 120 °C for 3 days. The mixture was then diluted with H_2_O (10 mL) and extracted with Et_2_O (3 × 5 mL). The combined organic layers were dried over anhydrous MgSO₄, filtered, and concentrated under reduced pressure. The product was purified by flash chromatography on silica gel using pentane:Et_2_O (5:1) to afford **P25** as a white solid (5.2 mg, 0.041 mmol, 28% yield, 97 % ee). Spectroscopic data matched with previous reported ones.^[20]^ ^1^H-NMR (400 MHz, CDCl_3_) δ, ppm: 4.81 (t, *J* = 6.0, 1H), 2.65 – 2.62 (m, 1H), 2.44 – 2.38 (m, 1H), 2.03 – 1.90 (m, 2H), 1.81 – 1.75 (m, 3H), 1.66 – 1.53 (m, 2H). ^13^C-NMR (101 MHz, CDCl_3_) δ, ppm: 179.1, 78.0, 39.1, 37.8, 28.2, 26.7, 18.6. ${[\alpha]}_{D}^{20}$= +4.9 (c = 0.233, CHCl_3_). HRMS (ESI+MS) *m/z* calculated for C_7_H_10_O_2_ [M+Na]^+^ 149.0573, found 149.0569.

**N-(7-oxo-6-oxabicyclo[3.2.1]octan-1-yl)benzamide (P26):** Lactone **P20** (15 mg, 1 equiv.) was dissolved in SOCl_2_ and the solution was stirred for 2 hours at room temperature. Then, SOCl_2_ was evaporated under reduced pressure and NaN_3_ (6.4 mg, 1.2 equiv) and anhydrous toluene (3 mL) were added, and the resulting mixture was left stirring for 3 hours at room temperature. After completion of reaction, 2 M NaHCO_3_ (10 mL) was added, and the aqueous solution was extracted with CH_2_Cl_2_ (3 x 5 mL). The combined organic phases were dried over anhydrous MgSO_4_, filtered, and concentrated under reduced pressure.^[21]^ The crude product was dissolved in THF (3 mL) and the solution was heated at 100°C for 1 hour. After that, a few drops of H_2_O, K_2_CO_3_ (51 mg, 4 equiv), and benzoyl chloride (13 μL, 1 equiv) were added, and the reaction was stirred overnight at room temperature. The mixture was diluted with H_2_O (5 mL) and it was extracted with AcOEt (3 x 10 mL). The combined organic phases were dried over anhydrous MgSO_4_, filtered, and concentrated under reduced pressure.^[22]^ The product was purified by flash chromatography on silica gel using hexane:EtOAc (10:1) to afford **P26** as a white solid (8 mg, 0.033 mmol, 37% yield). ^1^H-NMR (400 MHz, CDCl_3_) δ, ppm: 7.80 – 7.77 (m, 2H), 7.55 – 7.50 (m, 1H), 7.46 – 7.42 (m, 2H), 4.98 (t, *J* = 5.1 Hz, 1H), 2.76 – 2.71 (m, 2H), 2.62 – 2.53 (m, 1H), 2.07 – 2.02 (m, 1H), 1.97 – 1.90 (m, 1H), 1.83 – 1.84 (m, 2H), 1.65 – 1.60 (m, 2H). ^13^C-NMR (101 MHz, CDCl_3_) δ, ppm: 176.9, 167.0, 133.9, 132.1, 128.8, 127.1, 77.8, 59.4, 42.5, 31.9, 27.1, 18.2. ${[\alpha]}_{D}^{20}$= +25.95 (c = 0.133, CHCl_3_). HRMS (ESI-MS) m/z calculated for C_14_H_15_NO_3_ [M+Na]^+^ 268.0944, found 268.0949.

**dimethyl-3-hydroxycyclohexane-1,1-dicarboxylate (P27):** Lactone **P20** (15 mg, 1 equiv.) was dissolved in MeOH (3 mL) and 2 drops of conc. H_2_SO_4_ were added. The mixture was stirred and refluxed overnight. After that, H_2_O (5 mL) was added and the solution was extracted with EtOAc (3 x 5 mL). The combined organic phases were dried over anhydrous MgSO_4_, filtered, and concentrated under reduced pressure. The product was purified by flash chromatography on silica gel using hexane:EtOAc (10:1) to afford **P27** as a colorless oil (13 mg, 0.06 mmol, 68% yield, 98% ee). ^1^H-NMR (400 MHz, CDCl_3_) δ, ppm: 3.84 (ddd, *J* = 12.4, 8.7, 3.6 Hz, 1H), 3.72 (s, 3H), 3.72 (s, 3H), 2.44 – 2.40 (m, 1H), 2.13 – 2.09 (m, 1H), 1.86 – 1.74 (m, 5H), 1.46 – 1.33 (m, 2H). ^13^C-NMR (101 MHz, CDCl_3_) δ, ppm: 172.5, 171.8, 66.9, 55.0, 52.9, 52.8, 39.1, 33.9, 30.8, 20.0. ${[\alpha]}_{D}^{20}$= -10.2 (c = 0.384, CHCl_3_). HRMS (ESI-MS) m/z calculated for C_10_H_16_O_5_ [M+Na]^+^ 239.0890, found 239.0897.

**8. Sequential lactonization of (S17) by double addition of catalyst**

Substrate **S17** was subjected to the optimized catalytic conditions using **(*S,S*)-2^iPr^**, affording **P17**. After quenching the reaction with isopropanol and removing the solvent under reduced pressure, the crude mixture was dissolved in EtOAc (2 mL) and filtered through a silica plug to remove the catalyst. The solvent was then evaporated to dryness, and the resulting crude was resubmitted to the same catalytic conditions. Since product **P17** is chiral, both enantiomers of catalyst **2^iPr^** were tested to evaluate possible matched and mismatched effects between the chiral catalyst and the desymmetrized intermediate.

In both cases, the bilactone ***bis*-P17** was obtained as a mixture of three diastereomers. Analysis of the two reactions revealed that the second lactonization proceeds more efficiently (both in terms of diastereomeric ratio and enantiomeric excess) when using the same catalyst chirality as in the first step. Under matched conditions, a d.r. of 1:2:10 was observed, with the major diastereomer formed in >99.9% ee. In contrast, switching to the opposite catalyst chirality resulted in a slightly lower d.r. (1.6:5.5:10) and a modest decrease in ee for the major diastereomer (90%).

**Figure S1.** Double lactonization of substrate **S17** was used to evaluate the matched and mismatched effects of the chiral catalyst.

**9. Catalyst inhibition by cyclopentane dicarboxylic acid (S19)**

Dropwise addition of H_2_O_2_ to a mixture of substrate **S19** and catalyst **2^iPr^** in TFE resulted in no observable color change in the solution. Analysis of the reaction mixture showed neither formation of the desired product nor any significant decomposition, and the starting material was largely recovered. These observations suggested that the catalyst might be inhibited by chelation between **S19** and **2^iPr^**, preventing formation of the active catalytic species. To investigate this possibility, ESI-MS analysis of the crude mixture prior to H_2_O_2_ addition was performed, which revealed a detectable intermediate consistent with coordination between **S19** and the catalyst. This supports the hypothesis that the structure of this carboxylic acid promotes strong chelation to the metal center, thereby inhibiting catalysis.

ESI-MS before addition of H_2_O_2_


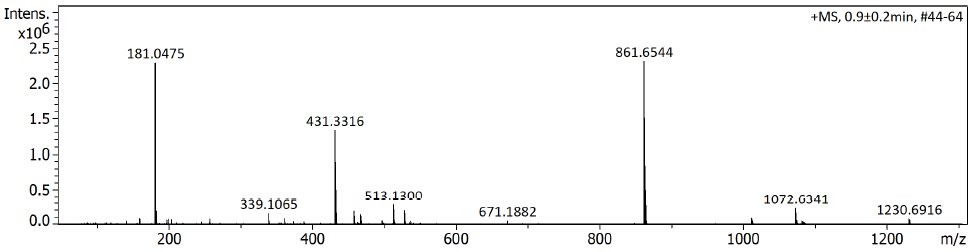

**Figure S2.** Inhibition of catalytic system via chelation

**10. Solid state structures by X-ray diffraction analysis**

10.1. Solid state structure of **P13** isolated using **(*R,R*)-2^iPr^**.

**Table S2.** Crystal data for **P13**. The absolute configuration could be determined by X-ray diffraction analysis.

| **Chemical formula** | C_10_H_14_O_4_ | | |  |
| --- | --- | --- | --- | --- |
| **Formula weight** | 198.21 g/mol | | |  |
| **Temperature** | 100(2) K | | |  |
| **Wavelength** | 0.71073 Å | | |  |
| **Crystal size** | 0.050 x 0.140 x 0.550 mm | | |  |
| **Crystal habit** | colorless needle | | |  |
| **Crystal system** | orthorhombic | | |  |
| **Space group** | P 21 21 21 | | |  |
| **Unit cell dimensions** | a = 6.8965(4) Å | | α = 90° |  |
|  | b = 7.5032(4) Å | | β = 90° |  |
|  | c = 18.7216(10) Å | | γ = 90° |  |
| **Volume** | 968.76(9) Å^3^ | |  |  |
| **Z** | 4 | | |  |
| **Density (calculated)** | 1.359 g/cm^3^ | | |  |
| **Absorption coefficient** | 0.105 mm^-1^ | | |  |
| **F(000)** | 424 | | |  |
| **Diffractometer** | D8 QUEST ECO three-circle diffractometer | | |  |
| **Radiation source** | Ceramic x-ray tube (Mo Kα, λ = 0.71073 Å) | | |  |
| **Theta range for data collection** | 3.15 to 38.61° | | |  |
| **Index ranges** | -12<=h<=12, -13<=k<=13, -32<=l<=32 | | |  |
| **Reflections collected** | 40366 | | |  |
| **Independent reflections** | 5453 [R(int) = 0.0249] | | |  |
| **Coverage of independent reflections** | 99.5% | | |  |
| **Absorption correction** | Multi-Scan | | |  |
| **Max. and min. transmission** | 0.9950 and 0.9450 | | |  |
| **Structure solution technique** | direct methods | | |  |
| **Structure solution program** | XT, VERSION 2018/2 | | |  |
| **Refinement method** | Full-matrix least-squares on F2 | | |  |
| **Refinement program** | SHELXL-2019/1 (Sheldrick, 2019) | | |  |
| **Function minimized** | Σ w(Fo2 - Fc2)2 | | |  |
| **Data / restraints / parameters** | 5453 / 0 / 134 | | |  |
| **Goodness-of-fit on F2** | 1.143 | | |  |
| **Δ/σmax** | 0.001 | | |  |
| **Final R indices** | 5290 data; I>2σ(I) | R1 = 0.0273, wR2 = 0.0693 | | |
|  | all data | R1 = 0.0287, wR2 = 0.0700 | | |
| **Weighting scheme** | w=1/[σ^2^(F_o_^2^)+(0.0342P)^2^+0.0771P] where P=(F_o_^2^+2F_c_^2^)/3 | | | |
| **Absolute structure parameter** | -0.02(11) | | | |
| **Largest diff. peak and hole** | 0.293 and -0.159 eÅ^-3^ | | | |
| **R.M.S. deviation from mean** | 0.038 eÅ^-3^ | | | |

10.2. Solid state structure of ***bis*-P16^syn-sym^** isolated using **(*R,R*)-2^iPr^**.

**Table S3.** Crystal data for ***bis*-P16^syn-sym^**.

| **Chemical formula** | C_9_H_12_O_4_ | | |  |
| --- | --- | --- | --- | --- |
| **Formula weight** | 184.19 g/mol | | |  |
| **Temperature** | 100(2) K | | |  |
| **Wavelength** | 0.71073 Å | | |  |
| **Crystal size** | 0.200 x 0.230 x 0.370 mm | | |  |
| **Crystal habit** | colorless block | | |  |
| **Crystal system** | tetragonal | | |  |
| **Space group** | P 41 21 2 | | |  |
| **Unit cell dimensions** | a = 6.24020(10) Å | | α = 90° |  |
|  | b = 6.24020(10) Å | | β = 90° |  |
|  | c = 44.5717(17) Å | | γ = 90° |  |
| **Volume** | 1735.63(9) Å^3^ | |  |  |
| **Z** | 8 | | |  |
| **Density (calculated)** | 1.410 g/cm^3^ | | |  |
| **Absorption coefficient** | 0.111 mm^-1^ | | |  |
| **F(000)** | 784 | | |  |
| **Diffractometer** | D8 QUEST ECO three-circle diffractometer | | |  |
| **Radiation source** | Ceramic x-ray tube (Mo Kα, λ = 0.71073 Å) | | |  |
| **Theta range for data collection** | 3.30 to 27.46° | | |  |
| **Index ranges** | -8<=h<=8, -8<=k<=8, -57<=l<=57 | | |  |
| **Reflections collected** | 49907 | | |  |
| **Independent reflections** | 1991 [R(int) = 0.0487] | | |  |
| **Coverage of independent reflections** | 99.9% | | |  |
| **Absorption correction** | Multi-Scan | | |  |
| **Max. and min. transmission** | 0.9780 and 0.9600 | | |  |
| **Structure solution technique** | direct methods | | |  |
| **Structure solution program** | XT, VERSION 2018/2 | | |  |
| **Refinement method** | Full-matrix least-squares on F2 | | |  |
| **Refinement program** | SHELXL-2019/1 (Sheldrick, 2019) | | |  |
| **Function minimized** | Σ w(Fo2 - Fc2)2 | | |  |
| **Data / restraints / parameters** | 1991 / 0 / 166 | | |  |
| **Goodness-of-fit on F2** | 1.110 | | |  |
| **Δ/σmax** | 0.001 | | |  |
| **Final R indices** | 1905 data; I>2σ(I) | R1 = 0.0307, wR2 = 0.0768 | | |
|  | all data | R1 = 0.0327, wR2 = 0.0777 | | |
| **Weighting scheme** | w=1/[σ^2^(F_o_^2^)+(0.0374P)^2^+0.5885P] where P=(F_o_^2^+2F_c_^2^)/3 | | | |
| **Absolute structure parameter** | 0.2(3) | | | |
| **Largest diff. peak and hole** | 0.233 and -0.180 eÅ^-3^ | | | |
| **R.M.S. deviation from mean** | 0.039 eÅ^-3^ | | | |

10.3 Solid state structure of **P17** isolated using **(*S,S*)-2^iPr^**.

**Table S4.** Crystal data for **P17**. The absolute configuration could be determined by X-ray diffraction analysis.

| **Chemical formula** | C_39_H_37_Cl_3_O_8_ | | |  |
| --- | --- | --- | --- | --- |
| **Formula weight** | 740.03 g/mol | | |  |
| **Temperature** | 100(2) K | | |  |
| **Wavelength** | 0.71073 Å | | |  |
| **Crystal size** | 0.060 x 0.060 x 0.320 mm | | |  |
| **Crystal habit** | colorless needle | | |  |
| **Crystal system** | orthorhombic | | |  |
| **Space group** | P 21 21 21 | | |  |
| **Unit cell dimensions** | a = 5.9306(9) Å | | α = 90° |  |
|  | b = 21.269(4) Å | | β = 90° |  |
|  | c = 28.869(4) Å | | γ = 90° |  |
| **Volume** | 3641.5(10) Å^3^ | |  |  |
| **Z** | 4 | | |  |
| **Density (calculated)** | 1.350 g/cm^3^ | | |  |
| **Absorption coefficient** | 0.304 mm^-1^ | | |  |
| **F(000)** | 1544 | | |  |
| **Diffractometer** | D8 QUEST ECO three-circle diffractometer | | |  |
| **Radiation source** | Ceramic x-ray tube (Mo Kα, λ = 0.71073 Å) | | |  |
| **Theta range for data collection** | 2.32 to 27.55° | | |  |
| **Index ranges** | -7<=h<=7, -27<=k<=22, -37<=l<=37 | | |  |
| **Reflections collected** | 30589 | | |  |
| **Independent reflections** | 6847 [R(int) = 0.0983] | | |  |
| **Coverage of independent reflections** | 91.1% | | |  |
| **Absorption correction** | Multi-Scan | | |  |
| **Max. and min. transmission** | 0.9820 and 0.9090 | | |  |
| **Structure solution technique** | direct methods | | |  |
| **Structure solution program** | XT, VERSION 2018/2 | | |  |
| **Refinement method** | Full-matrix least-squares on F2 | | |  |
| **Refinement program** | SHELXL-2019/1 (Sheldrick, 2019) | | |  |
| **Function minimized** | Σ w(Fo2 - Fc2)2 | | |  |
| **Data / restraints / parameters** | 6847 / 234 / 423 | | |  |
| **Goodness-of-fit on F2** | 1.121 | | |  |
| **Final R indices** | 5203 data; I>2σ(I) | R1 = 0.1584, wR2 = 0.3737 | | |
|  | all data | R1 = 0.1871, wR2 = 0.3901 | | |
| **Weighting scheme** | w=1/[σ^2^(F_o_^2^)+(0.1338P)^2^+50.9096P] where P=(F_o_^2^+2F_c_^2^)/3 | | | |
| **Absolute structure parameter** | 0.07(6) | | | |
| **Largest diff. peak and hole** | 1.405 and -0.733 eÅ^-3^ | | | |
| **R.M.S. deviation from mean** | 0.177 eÅ^-3^ | | | |

10.4. Solid state structure of ***bis*-P18** isolated using **(*R,R*)-2^iPr^**.

**Table S5.** Crystal data for ***bis*-P18**.

| **Chemical formula** | **C_11_H_16_O_4_** | | |  |
| --- | --- | --- | --- | --- |
| **Formula weight** | **212.24 g/mol** | | |  |
| **Temperature** | **100(2) K** | | |  |
| **Wavelength** | **0.71073 Å** | | |  |
| **Crystal size** | **0.140 x 0.150 x 0.290 mm** | | |  |
| **Crystal habit** | **colorless prism** | | |  |
| **Crystal system** | **orthorhombic** | | |  |
| **Space group** | **P b c a** | | |  |
| **Unit cell dimensions** | **a = 10.8691(10) Å** | | **α = 90°** |  |
|  | **b = 10.9702(10) Å** | | **β = 90°** |  |
|  | **c = 18.3816(16) Å** | | **γ = 90°** |  |
| **Volume** | **2191.8(3) Å^3^** | |  |  |
| **Z** | **8** | | |  |
| **Density (calculated)** | **1.286 g/cm^3^** | | |  |
| **Absorption coefficient** | **0.097 mm^-1^** | | |  |
| **F(000)** | **912** | | |  |
| **Diffractometer** | **D8 QUEST ECO three-circle diffractometer** | | |  |
| **Radiation source** | **Ceramic x-ray tube (Mo Kα, λ = 0.71073 Å)** | | |  |
| **Theta range for data collection** | **3.45 to 33.17°** | | |  |
| **Index ranges** | **-16<=h<=16, -16<=k<=16, -28<=l<=28** | | |  |
| **Reflections collected** | **54315** | | |  |
| **Independent reflections** | **4168 [R(int) = 0.0766]** | | |  |
| **Coverage of independent reflections** | **99.7%** | | |  |
| **Absorption correction** | **Multi-Scan** | | |  |
| **Max. and min. transmission** | **0.9870 and 0.9720** | | |  |
| **Structure solution technique** | **direct methods** | | |  |
| **Structure solution program** | **XT, VERSION 2018/2** | | |  |
| **Refinement method** | **Full-matrix least-squares on F2** | | |  |
| **Refinement program** | **SHELXL-2019/1 (Sheldrick, 2019)** | | |  |
| **Function minimized** | **Σ w(Fo2 - Fc2)2** | | |  |
| **Data / restraints / parameters** | **4168 / 0 / 200** | | |  |
| **Goodness-of-fit on F2** | **1.087** | | |  |
| **Final R indices** | 3295 data; I>2σ(I) | R1 = 0.0534, wR2 = 0.1143 | | |
|  | all data | R1 = 0.0753, wR2 = 0.1229 | | |
| **Weighting scheme** | w=1/[σ^2^(F_o_^2^)+(0.0500P)^2^+0.9263P] where P=(F_o_^2^+2F_c_^2^)/3 | | | |
| **Largest diff. peak and hole** | 0.450 and -0.227 eÅ^-3^ | | | |
| **R.M.S. deviation from mean** | 0.056 eÅ^-3^ | | | |

10.5. Solid state structure of **P20** isolated using **(*R,R*)-2^iPr^**.

**Table S6.** Crystal data for **P20**. The absolute configuration could be determined by X-ray diffraction analysis.

| **Chemical formula** | **C_8_H_10_O_4_** | | |  |
| --- | --- | --- | --- | --- |
| **Formula weight** | **170.16 g/mol** | | |  |
| **Temperature** | **100(2) K** | | |  |
| **Wavelength** | **0.71073 Å** | | |  |
| **Crystal size** | **0.060 x 0.240 x 0.270 mm** | | |  |
| **Crystal habit** | **colorless plate** | | |  |
| **Crystal system** | **orthorhombic** | | |  |
| **Space group** | **P 21 21 21** | | |  |
| **Unit cell dimensions** | **a = 11.1425(5) Å** | | **α = 90°** |  |
|  | **b = 11.6458(5) Å** | | **β = 90°** |  |
|  | **c = 12.1512(4) Å** | | **γ = 90°** |  |
| **Volume** | **1576.78(11) Å^3^** | |  |  |
| **Z** | **8** | | |  |
| **Density (calculated)** | **1.434 g/cm^3^** | | |  |
| **Absorption coefficient** | **0.116 mm^-1^** | | |  |
| **F(000)** | **720** | | |  |
| **Diffractometer** | **D8 QUEST ECO three-circle diffractometer** | | |  |
| **Radiation source** | **Ceramic x-ray tube (Mo Kα, λ = 0.71073 Å)** | | |  |
| **Theta range for data collection** | **3.04 to 28.33°** | | |  |
| **Index ranges** | **-14<=h<=14, -15<=k<=15, -16<=l<=16** | | |  |
| **Reflections collected** | **92087** | | |  |
| **Independent reflections** | **3916 [R(int) = 0.0621]** | | |  |
| **Coverage of independent reflections** | **99.7%** | | |  |
| **Absorption correction** | **Multi-Scan** | | |  |
| **Max. and min. transmission** | **0.9930 and 0.9690** | | |  |
| **Structure solution technique** | **direct methods** | | |  |
| **Structure solution program** | **XT, VERSION 2018/2** | | |  |
| **Refinement method** | **Full-matrix least-squares on F2** | | |  |
| **Refinement program** | **SHELXL-2019/1 (Sheldrick, 2019)** | | |  |
| **Function minimized** | **Σ w(Fo2 - Fc2)2** | | |  |
| **Data / restraints / parameters** | **3916 / 0 / 297** | | |  |
| **Goodness-of-fit on F2** | **1.150** | | |  |
| **Final R indices** | 3740 data; I>2σ(I) | R1 = 0.0368, wR2 = 0.0820 | | |
|  | all data | R1 = 0.0401, wR2 = 0.0834 | | |
| **Weighting scheme** | w=1/[σ^2^(F_o_^2^)+(0.0385P)^2^+0.4850P] where P=(F_o_^2^+2F_c_^2^)/3 | | | |
| **Absolute structure parameter** | -0.2(2) | | | |
| **Largest diff. peak and hole** | 0.306 and -0.187 eÅ^-3^ | | | |
| **R.M.S. deviation from mean** | 0.045 eÅ^-3^ | | | |

10.6. Solid state structure of **P24**.

**Table S7.** Crystal data for **P24**. Product was crystallized in racemic form.

| **Chemical formula** | C_9_H_12_O_4_ | | |  |
| --- | --- | --- | --- | --- |
| **Formula weight** | 184.19 g/mol | | |  |
| **Temperature** | 100(2) K | | |  |
| **Wavelength** | 0.71073 Å | | |  |
| **Crystal size** | 0.060 x 0.130 x 0.220 mm | | |  |
| **Crystal habit** | colorless plate | | |  |
| **Crystal system** | orthorhombic | | |  |
| **Space group** | P b c n | | |  |
| **Unit cell dimensions** | a = 13.493(3) Å | | α = 90° |  |
|  | b = 12.573(3) Å | | β = 90° |  |
|  | c = 10.038(3) Å | | γ = 90° |  |
| **Volume** | 1702.9(8) Å^3^ | |  |  |
| **Z** | 8 | | |  |
| **Density (calculated)** | 1.437 g/cm^3^ | | |  |
| **Absorption coefficient** | 0.113 mm^-1^ | | |  |
| **F(000)** | 784 | | |  |
| **Diffractometer** | D8 QUEST ECO three-circle diffractometer | | |  |
| **Radiation source** | Ceramic x-ray tube (Mo Kα, λ = 0.71073 Å) | | |  |
| **Theta range for data collection** | 3.00 to 27.58° | | |  |
| **Index ranges** | -17<=h<=17, -16<=k<=16, -13<=l<=13 | | |  |
| **Reflections collected** | 30680 | | |  |
| **Independent reflections** | 1969 [R(int) = 0.0935] | | |  |
| **Coverage of independent reflections** | 99.9% | | |  |
| **Absorption correction** | Multi-Scan | | |  |
| **Max. and min. transmission** | 0.9930 and 0.9760 | | |  |
| **Structure solution technique** | direct methods | | |  |
| **Structure solution program** | XT, VERSION 2018/2 | | |  |
| **Refinement method** | Full-matrix least-squares on F2 | | |  |
| **Refinement program** | SHELXL-2019/1 (Sheldrick, 2019) | | |  |
| **Function minimized** | Σ w(Fo2 - Fc2)2 | | |  |
| **Data / restraints / parameters** | 1969 / 0 / 122 | | |  |
| **Goodness-of-fit on F2** | 1.077 | | |  |
| **Final R indices** | 1505 data; I>2σ(I) | R1 = 0.0586, wR2 = 0.1387 | | |
|  | all data | R1 = 0.0828, wR2 = 0.1537 | | |
| **Weighting scheme** | w=1/[σ^2^(F_o_^2^)+(0.0673P)^2^+2.0137P] where P=(F_o_^2^+2F_c_^2^)/3 | | | |
| **Largest diff. peak and hole** | 0.970 and -0.285 eÅ^-3^ | | | |
| **R.M.S. deviation from mean** | 0.074 eÅ^-3^ | | | |

**11. ^1^H and ^13^C-NMR of the substrates**

^1^H-NMR of **S1** in CDCl_3_

^13^C-NMR of **S1** in CDCl_3_

^1^H-NMR of **S2** in CDCl_3_

^13^C-NMR of **S1** in CDCl_3_

^1^H-NMR of **S3a** in CDCl_3_

^13^C-NMR of **S3a** in CDCl_3_

^1^H-NMR of **S3** in CDCl_3_

^13^C-NMR of **S3** in CDCl_3_

{^1^H}^19^F-NMR of **S3** in CDCl_3_

^1^H-NMR of **S4** in CDCl_3_

^13^C-NMR of **S4** in CDCl_3_

^1^H-NMR of **S5a** in CDCl_3_

^13^C-NMR of **S5a** in CDCl_3_

^1^H-NMR of **S5** in CDCl_3_

^13^C-NMR of **S5** in CDCl_3_

^1^H-NMR of **S6a** in CDCl_3_

^13^C-NMR of **S6a** in CDCl_3_

^1^H-NMR of **S6** in CDCl_3_

^13^C-NMR of **S6** in CDCl_3_

^1^H-NMR of **S7a** in CDCl_3_

^13^C-NMR of **S7a** in CDCl_3_

^1^H-NMR of **S7** in CDCl_3_

^13^C-NMR of **S7** in CDCl_3_

^1^H-NMR of **S8a** in CDCl_3_

^13^C-NMR of **S8a** in CDCl_3_

^1^H-NMR of **S8** in CDCl_3_

^13^C-NMR of **S8** in CDCl_3_

^1^H-NMR of **S9a** in CDCl_3_

^13^C-NMR of **S9a** in CDCl_3_

^1^H-NMR of **S9** in CDCl_3_

^13^C-NMR of **S9** in CDCl_3_

^1^H-NMR of **S10** in CDCl_3_

^13^C-NMR of **S10** in CDCl_3_

{^1^H}^19^F-NMR of **S10** in CDCl_3_

^1^H-NMR of **S11** in CDCl_3_

^13^C-NMR of **S11** in CDCl_3_

^1^H-NMR of **S12** in CDCl_3_

^13^C-NMR of **S12** in CDCl_3_

^1^H-NMR of **S13** in CDCl_3_

^13^C-NMR of **S13** in CDCl_3_

^1^H-NMR of **S14** in CDCl_3_

^13^C-NMR of **S14** in CDCl_3_

^1^H-NMR of **S15** in CDCl_3_

^13^C-NMR of **S15** in CDCl_3_

^1^H-NMR of **S16** in CD₃OD

^13^C-NMR of **S16** in CD₃OD

^1^H-NMR of **S17** in CD₃OD

^13^C-NMR of **S17** in CD₃OD

^1^H-NMR of **S18** in CD₃OD

^13^C-NMR of **S18** in CD₃OD

^1^H-NMR of **S19** in CD₃OD

^13^C-NMR of **S19** in CD₃OD

^1^H-NMR of **S21a** in CDCl_3_

^13^C-NMR of **S21a** in CDCl_3_

^1^H-NMR of **S21** in CD₃OD

^13^C-NMR of **S21** in CD₃OD

^1^H-NMR of **S22a** (mixture of isomers) in CDCl_3_

^13^C-NMR of **S22a** (mixture of isomers) in CDCl_3_

^1^H-NMR of **S22** in CD₃OD

^13^C-NMR of **S22** in CD₃OD

^1^H-NMR of **S23a** in CDCl_3_

^13^C-NMR of **S23a** in CDCl_3_

^1^H-NMR of **S23b** in CDCl_3_

^13^C-NMR of **S23b** in CDCl_3_

^1^H-NMR of **S23** in CD₃OD

^13^C-NMR of **S23** in CD₃OD

^1^H-NMR of **S24** in CD₃OD

^13^C-NMR of **S24** in CD₃OD

**12. ^1^H and ^13^C-NMR of the isolated lactones**

^1^H-NMR of **P1** in CDCl_3_

^13^C-NMR of **P1** in CDCl_3_

^1^H-NMR of **P2** in CDCl_3_

^13^C-NMR of **P2** in CDCl_3_

^1^H-NMR of **P3** in CDCl_3_

^13^C-NMR of **P3** in CDCl_3_

{^1^H}^19^F-NMR of **P3** in CDCl_3_

^1^H-NMR of **P4** (*major diastereomer*) in CDCl_3_

^13^C-NMR of **P4** (*major diastereomer*) in CDCl_3_

^1^H-NMR of **P4** (*mixture of both diastereomers*) in CDCl_3_

^13^C-NMR of **P4** (*mixture of both diastereomers*) in CDCl_3_

^1^H-NMR of **P5** in CDCl_3_

^13^C-NMR of **P5** in CDCl_3_

^1^H-NMR of **P6** in CDCl_3_

^13^C-NMR of **P6** in CDCl_3_

^1^H-NMR of **P7** (*major diastereomer*) in CDCl_3_

^13^C-NMR of **P7** (*major diastereomer*) in CDCl_3_

^1^H-NMR of **P7** (*mixture of both diastereomers*) in CDCl_3_

^13^C-NMR of **P7** (*mixture of both diastereomers*) in CDCl_3_

^1^H-NMR of **δ-P7** in CDCl_3_

^13^C-NMR of **δ-P7** in CDCl_3_

^1^H-NMR of **P8** (*major diastereomer*) in CDCl_3_

^13^C-NMR of **P8** (*major diastereomer*) in CDCl_3_

^1^H-NMR of **P8** (*mixture of both diastereomers*) in CDCl_3_

^13^C -NMR of **P8** (*mixture of both diastereomers*) in CDCl_3_

^1^H-NMR of **δ-P9** in CDCl_3_

^13^C-NMR of **δ-P9** in CDCl_3_

^1^H-NMR of **P9** (*major diastereomer*) in CDCl_3_

^13^C-NMR of **P9** (*major diastereomer*) in CDCl_3_

^1^H-NMR of **P9** (*mixture of both diastereomers*) in CDCl_3_

^1^H-NMR of **δ-P9** (*major diastereomer*) in CDCl_3_

^13^C-NMR of **δ-P9** (*major diastereomer*) in CDCl_3_

^1^H-NMR of **δ-P9** (*mixture of both diastereomers*) in CDCl_3_

^1^H-NMR of **P10** (*mixture of both diastereomers*) in CD_2_Cl_2_

{^1^H}^19^F -NMR of **δ-P10** (*mixture of both diastereomers*) in CD_2_Cl_2_

^1^H-NMR of **δ-P10** (*major diastereomer*) in CDCl_3_

^13^C-NMR of **δ-P10** (*major diastereomer*) in CDCl_3_

{^1^H}^19^F -NMR of **δ-P10** in CDCl_3_

^1^H-NMR of **P11** in CDCl_3_

^13^C-NMR of **P11** in CDCl_3_

^1^H-NMR of **P12** in CDCl_3_

^13^C-NMR of **P12** in CDCl_3_

^1^H-NMR of **P13** in CDCl_3_

^13^C-NMR of **P13** in CDCl_3_

^1^H-NMR of **P14** in CDCl_3_

^13^C-NMR of **P14** in CDCl_3_

^1^H-NMR of **P15** (*mixture with the substrate* ***S15***) in CDCl_3_

^13^C-NMR of **P15** (*mixture with the substrate* ***S15***) in CDCl_3_

^1^H-NMR of ***bis*-P16^syn-sym^** in CDCl_3_

^13^C-NMR of ***bis*-P16^syn-sym^** in CDCl_3_

^1^H-NMR of ***bis*-P16^unsym^** in CDCl_3_

^1^H-NMR of ***bis*-P16^anti-sym^** in CDCl_3_

^1^H-NMR of **P17** (*major diastereomer*) in CDCl_3_

^13^C-NMR of **P17** (*major diastereomer*) in CDCl_3_

^1^H-NMR of **P17** (*mixture of both diastereomers*) in CDCl_3_

^1^H-NMR of ***bis*-P17** in CDCl_3_

^13^C-NMR of ***bis*-P17** in CDCl_3_

^1^H-^13^C HMBC NMR of ***bis*-P17** in CDCl_3_

^1^H-NMR of ***bis*-P18** in CDCl_3_

^13^C-NMR of ***bis*-P18** in CDCl_3_

^1^H-NMR of **P20** in CDCl_3_

^13^C-NMR of **P20** in CDCl_3_

^1^H-NMR of **P24** in CDCl_3_

^13^C-NMR of **P24** in CDCl_3_

**13. ^1^H and ^13^C-NMR of the product elaboration**

^1^H-NMR of **P25** in CDCl_3_

^13^C-NMR of **P25** in CDCl_3_

^1^H-NMR of **P26** in CDCl_3_

^13^C-NMR of **P26** in CDCl_3_

^1^H-NMR of **P27** in CDCl_3_

^1^H-NMR of **P27** in CDCl_3_

**14. ^1^H and ^13^C-NMR of the derivatized lactones**

^1^H-NMR of **P7^unsym^** in CDCl_3_

^13^C-NMR of **P7^unsym^** in CDCl_3_

^1^H-^13^C HSQC NMR of **P7^unsym^** in CDCl_3_

^1^H-NMR of **P8^unsym^** in CDCl_3_

^13^C-NMR of **P8^unsym^** in CDCl_3_

^1^H-^13^C HSQC NMR of **P8^unsym^** in CDCl_3_

^1^H-^13^C HMBC NMR of **P8^unsym^** in CDCl_3_

^1^H-NMR of **P12^sym^** in CDCl_3_

^13^C-NMR of **P12^sym^** in CDCl_3_

^1^H-NMR of **P13^unsym^** in CDCl_3_

^13^C-NMR of **P13^unsym^** in CDCl_3_

^1^H-NMR of **P13^unsym^** in C_6_D_6_

^13^C-NMR of **P13^unsym^** in C_6_D_6_

^1^H-NMR of **P21^deriv^** in CDCl_3_

^13^C-NMR of **P21^deriv^** in CDCl_3_

^1^H-^13^C HMBC NMR of **P21^deriv^** in CDCl_3_

^1^H-NMR of **P22^deriv^** (*mixture of both diastereomers*) in CD_2_Cl_2_

^13^C-NMR of **P22^deriv^** (*mixture of both diastereomers*) in CD_2_Cl_2_

^1^H-^13^C HMBC NMR of **P22^deriv^** in CD_2_Cl_2_

^1^H-NMR of **P23^deriv^** in CD_2_Cl_2_

^13^C-NMR of **P23^deriv^** in CD_2_Cl_2_

^1^H-NMR of **P24^deriv^** in CDCl_3_

^13^C-NMR of **P24^deriv^** in CDCl_3_

**15. SFC and GC traces**

Products **P7**, **P8**, **P12**, **P13**, **P21**, **P22**, **P23** and **P24** were further functionalized to measure the enantioselectivities by SFC analysis.

**Derivatization of P7**

A round-bottom flask equipped with a septum and under a nitrogen atmosphere was charged with the major diastereomerically pure **P7** (55 mg, 0.24 mmol, 1 equiv.) and dry THF (5 mL). Lithium aluminium hydride (28 mg, 0.72 mmol, 3 equiv.) was added slowly at 0 °C, and the resulting mixture was stirred overnight at room temperature. The reaction was quenched carefully with a minimal amount of 2 M NaOH at 0 °C. After filtration through Celite^®^, the organic layer was dried over anhydrous Na_2_CO_3_, filtered, and concentrated under reduced pressure to afford **P7^triol^** (27 mg, 0.14 mmol, 59% yield). The triol was identified by GC-MS and used immediately without further purification. Followed a slight modification of a reported procedure,^[23]^ the crude triol was dissolved in toluene, and phenylboronic acid (14 mg, 0.12 mmol, 0.9 equiv.) was added. The mixture was stirred under reflux with a Dean-Stark apparatus for 2 h, leading to full conversion of **P7^triol^** as observed by GC, giving symmetric and unsymmetric products in a 1:9 ratio. The reaction mixture was concentrated under reduced pressure and directly subjected to flash chromatography on silica gel using DCM:MeOH (98:2) to afford the derivatized product **P7^unsym^** as a colorless oil (12 mg, 0.04 mmol, 29% yield, 95% ee). Characterization of the product has been done with the racemic form due to higher amount of product since separation of diastereomers in the first step is not necessary. ^1^H-NMR (400 MHz, CDCl_3_) δ, ppm: 7.81 – 7.78 (m, 2H), 7.45 – 7.41 (m, 1H), 7.35 (t, *J* = 7.1 Hz, 2H), 4.06 (d, *J* = 11.2 Hz, 1H), 3.96 (d, *J* = 11.2 Hz, 1H), 3.88 (s, 1H), 3.73 – 3.68 (m, 1H), 1.53 – 1.42 (m, 6H), 1.34 – 1.28 (m, 2H), 0.95 – 0.89 (m, 6H). ^13^C-NMR (101 MHz, CDCl_3_) δ, ppm: 134.0, 133.4, 130.8, 127.7, 70.7, 69.9, 69.8, 38.7, 32.4, 31.9, 25.3, 23.6, 14.2, 10.0. HRMS (ESI-MS) m/z calculated for C_17_H_27_B_1_O_3_ [M+Na]^+^ 313.1949, found 313.1948.

**Derivatization of P8**

A round-bottom flask equipped with a septum and under a nitrogen atmosphere was charged with the major diastereomerically pure **P8** (33 mg, 0.13 mmol, 1 equiv.) and dry THF (5 mL). Lithium aluminium hydride (15 mg, 0.39 mmol, 3 equiv.) was added slowly at 0 °C, and the resulting mixture was stirred overnight at room temperature. The reaction was quenched carefully with a minimal amount of 2 M NaOH at 0 °C. After filtration through Celite^®^, the organic layer was dried over anhydrous Na_2_CO_3,_ filtered, and concentrated under reduced pressure to afford **P8^triol^** (24 mg, 0.10 mmol, 80% yield). The triol was identified by GC-MS and used immediately without further purification. Followed a slight modification of a reported procedure,^[23]^ the crude triol was dissolved in toluene, and phenylboronic acid (11 mg, 0.09 mmol, 0.9 equiv.) was added. The mixture was stirred under reflux with a Dean-Stark apparatus for 2 h, leading to full conversion of **P8^triol^** as observed by GC, giving symmetric and unsymmetric products in a 2:3 ratio. The reaction mixture was concentrated under reduced pressure and directly subjected to flash chromatography on silica gel using DCM:MeOH (98:2) to afford the derivatized product **P8^unsym^** as a colorless oil (8 mg, 0.03 mmol, 24% yield, 92% ee). Characterization of the product has been done with the racemic form due to higher amount of product since separation of diastereomers in the first step is not necessary. ^1^H-NMR (400 MHz, CDCl_3_) δ, ppm: 7.79 (d, *J* = 6.6 Hz, 2H), 7.45 – 7.40 (m, 1H), 7.35 (t, *J* = 7.2 Hz, 2H), 4.05 (d, *J* = 12.2 Hz, 1H), 3.95 (d, *J* = 12.2 Hz, 1H), 3.87 (s, 2H), 3.60 – 3.57 (m, 1H), 1.67 – 1.61 (m, 1H), 1.54 – 1.39 (m, 5H), 1.24 – 1.16 (m, 1H), 1.14 – 1.05 (m, 1H), 0.92 – 0.88 (m, 12H). ^13^C-NMR (101 MHz, CDCl_3_) δ, ppm: 134.0, 133.0, 130.8, 127.7, 72.9, 70.8, 69.8, 35.7, 35.3, 31.9, 29.7, 28.8, 22.8, 22.7, 18.6, 17.3. HRMS (ESI-MS) m/z calculated for C_19_H_31_B_1_O_3_ [M+Na]^+^ 341.2262, found 341.2258.

**Derivatization of P12**

A round-bottom flask equipped with a septum and under a nitrogen atmosphere was charged with the pure **P12** (50 mg, 0.27 mmol, 1 equiv.) and dry THF (5 mL). Lithium aluminium hydride (31 mg, 0.81 mmol, 3 equiv.) was added slowly at 0 °C, and the resulting mixture was stirred overnight at room temperature. The reaction was quenched carefully with a minimal amount of 2 M NaOH at 0 °C. After filtration through Celite^®^, the organic layer was dried over anhydrous Na_2_CO_3,_ filtered, and concentrated under reduced pressure to afford **P12^triol^** (30 mg, 0.19 mmol, 69% yield). The triol was identified by GC-MS and used immediately without further purification. Followed a slight modification of a reported procedure,^[23]^ the crude triol was dissolved in toluene, and phenylboronic acid (23 mg, 0.17 mmol, 0.9 equiv.) was added. The mixture was stirred under reflux with a Dean-Stark apparatus for 2 h, leading to full conversion of **P12^triol^** as observed by GC, giving symmetric and unsymmetric products in a 10:1 ratio. The reaction mixture was concentrated under reduced pressure and directly subjected to flash chromatography on silica gel using DCM:MeOH (98:2) to afford the derivatized product **P12^sym^**  as a colorless oil (10 mg, 0.04 mmol, 22% yield, 99% ee). **ee% matches perfectly with the one measured with the chiral GC so the methodology is valid to measure the enantioselectivities.** ^1^H-NMR (400 MHz, CDCl_3_) δ, ppm: 7.78 (dd, *J* = 8.0, 1.4 Hz, 2H), 7.44 – 7.40 (m, 1H), 7.35 (t, *J* = 7.2 Hz, 2H), 3.94 (s, 2H), 3.84 (s, 2H), 3.88 – 3.81 (m, 1H), 2.00 – 1.96 (m, 1H), 1.86 (ddd, *J* = 13.1, 4.1, 2.0 Hz, 1H), 1.76 (dt, *J* = 13.7, 4.3 Hz, 1H), 1.66 – 1.61 (m, 1H), 1.52 – 1.46 (m, 1H), 1.46 – 1.42 (m, 1H), 1.36 – 1.29 (m, 1H), 1.24 – 1.18 (m, 1H). ^13^C-NMR (101 MHz, CDCl_3_) δ, ppm: 134.0, 133.3, 130.9, 127.7, 72.8, 68.5, 66.8, 39.4, 36.2, 35.6, 29.7, 19.5. ${[\alpha]}_{D}^{20}$= +3.84 (c = 0.36, CHCl_3_). HRMS (ESI-MS) m/z calculated for C_14_H_19_B_1_O_3_ [M+Na]^+^ 269.1322, found 269.1328.

**Derivatization of P13**

A round-bottom flask equipped with a septum and under a nitrogen atmosphere was charged with the pure **P13** (31 mg, 0.16 mmol, 1 equiv.) and dry THF (5 mL). Lithium aluminium hydride (18 mg, 0.48 mmol, 3 equiv.) was added slowly at 0 °C, and the resulting mixture was stirred overnight at room temperature. The reaction was quenched carefully with a minimal amount of 2 M NaOH at 0 °C. After filtration through Celite^®^, the organic layer was dried over anhydrous Na_2_CO_3,_  filtered, and concentrated under reduced pressure to afford **P13^triol^** (20 mg, 0.12 mmol, 73% yield). The triol was identified by GC-MS and used immediately without further purification. Followed a slight modification of a reported procedure,^[23]^ the crude triol was dissolved in toluene, and phenylboronic acid (12 mg, 0.11 mmol, 0.9 equiv.) was added. The mixture was stirred under reflux with a Dean-Stark apparatus for 2 h, leading to full conversion of **P7^triol^** as observed by GC, giving symmetric and unsymmetric products in a 3:10 ratio. The reaction mixture was concentrated under reduced pressure and directly subjected to flash chromatography on silica gel using DCM:MeOH (98:2) to afford the derivatized product **P13^unsym^** as a colorless oil (10 mg, 0.038 mmol, 33% yield, 99% ee). ^1^H-NMR (400 MHz, CDCl_3_) δ, ppm: 7.80 – 7.77 (m, 2H), 7.45 – 7.40 (m, 1H), 7.35 (t, *J* = 7.1 Hz, 2H), 3.95 – 3.83 (m, 3H), 3.81 (s, 2H), 2.03 – 1.99 (m, 1H), 1.85 – 1.79 (m, 2H), 1.76 – 1.70 (m, 1H), 1.67 – 1.63 (m, 1H), 1.56 – 1.49 (m, 1H), 1.45 – 1.34 (m, 3H), 1.30 – 1.24 (m, 1H). ^13^C-NMR (101 MHz, CDCl_3_) δ, ppm: 134.0, 133.4, 130.8, 127.7, 70.7, 69.9, 69.8, 38.7, 32.4, 31.9, 25.3, 23.6, 14.2, 10.0. ${[\alpha]}_{D}^{20}$= +16.30 (c = 0.457, CHCl_3_). HRMS (ESI-MS) m/z calculated for C_15_H_21_B_1_O_3_ [M+Na]^+^ 281.1479, found 281.1479.

**For better resolution and elucidation of the structure performed NMR analysis in C_6_D_6_.** ^1^H-NMR (400 MHz, C_6_D_6_) δ, ppm: 8.26 – 8.24 (m, 2H), 7.32 – 8.28 (m, 3H), 3.59 – 3.54 (dd, *J* = 20.0 Hz, *J* = 12.0 Hz, 2H), 3.48 (s, 2H), 3.35 (tt, *J* = 9.7, 2.5 Hz, 1H), 1.63 – 1.57 (m, 1H), 1.49 – 1.31 (m, 3H), 1.25 – 1.11 (m, 3H), 1.00 – 0.92 (m, 1H), 0.86 – 0.82 (m, 1H). ^13^C-NMR (101 MHz, C_6_D_6_) δ, ppm: 134.6, 133.8, 131.2, 128.2, 71.8, 71.1, 68.4, 42.3, 39.8, 35.3, 32.9, 26.5, 22.5.

**Derivatization of P21**

**P21^deriv^:** Lactone **P21^deriv^** was isolated using **(S,S)-** **2^H^** on a 0.375 mmol scale. After completion of the catalysis, the crude reaction mixture was evaporated to complete dryness and passed through a short silica plug with eluent EtOAc. The filtrate was concentrated under reduced pressure and subjected to esterification using TMSCN₂ (0.3 mL, 1.87 mmol, 5 equiv. relative to **S21**) in a MeOH:toluene (1:1) mixture. The reaction was stirred overnight at room temperature. After concentration under reduced pressure, the crude mixture was directly loaded onto a chromatographic column and purified by flash chromatography. chromatography on silica gel using hexane:EtOAc (25:1) to afford **P21^deriv^** as white solid (8 mg, 0.033 mmol, 9% yield over two steps, 41% ee). ^1^H-NMR (400 MHz, CDCl_3_) δ, ppm: 4.93 (d, *J* = 6.6 Hz, 1H), 3.78 (s, 3H), 2.94 (ddd, *J* = 11.5, 6.6, 2.9 Hz, 1H), 2.34 – 2.29 (m, 1H), 1.97 – 1.91 (dt, *J* = 14.3, 5.4 Hz, 1H), 1.86 (d, *J* = 11.5 Hz, 1H), 1.75 (td, *J* = 13.1, 5.7 Hz, 2H), 1.38 – 1.35 (m, 1H), 0.93 (s, 9H). ^13^C-NMR (101 MHz, CDCl_3_) δ, ppm: 174.1, 170.2, 77.2, 53.0, 52.9, 49.0, 42.3, 32.5, 28.7, 28.0, 20.2. ${[\alpha]}_{D}^{20}$= - 19.5 (c = 0.296, CHCl_3_). HRMS (ESI-MS) m/z calculated for C_13_H_20_O_4_ [M+H]^+^ 263.1254, found 263.1252.

**Derivatization of P22**

**P22^deriv^:** Lactone **P22^deriv^** was isolated using **(S,S)-2^iPr^** on a 0.269 mmol scale. After completion of the catalysis, the reaction mixture was evaporated to complete dryness, dissolved in DCM (2 mL), and treated with phenol (76 mg, 3 equiv. relative to **S22**) and EDC·HCl (155 mg, 3 equiv. relative to **S22**). The mixture was stirred overnight at room temperature. After concentration under reduced pressure, the crude mixture was directly loaded onto a chromatographic column and purified by flash chromatography on silica gel using hexane:EtOAc (25:1) to afford mixture of two isomers of the esterified derivatized product **P22^deriv^** as a white solid (3 mg, 0.012 mmol, 4% yield over two steps, >99.9% ee). *Spectroscopical data for the minor diastereomer; please note that, due to overlap with signals from the major isomer, only the following resonances could be unambiguously assigned*  ^1^H-NMR (400 MHz, CD_2_Cl_2_) δ, ppm: 7.43 – 7.38 (m, 2H), 7.29 – 7.25 (m, 1H), 7.13 – 7.09 (m, 2H), 4.64 (d, *J* = 6.4 Hz, 1H), 3.08 (ddd, *J* = 11.6, 6.4, 2.8 Hz, 1H), 1.06 (d, *J* = 6.8 Hz, 3H, major). *Spectroscopical data for the major diastereomer:* ^1^H-NMR (400 MHz, CD_2_Cl_2_) δ, ppm: 7.43 – 7.38 (m, 2H), 7.29 – 7.25 (m, 1H), 7.13 – 7.09 (m, 2H), 4.73 (dd, *J* = 6.2, 4.1 Hz, 1H), 2.85 (ddd, *J* = 11.9, 6.2, 2.9 Hz, 1H,), 2.33 (d, *J* = 11.9 Hz, 1H), 2.06 – 1.90 (m, 2H), 1.70 – 1.62 (m, 1H), 1.06 (d, *J* = 7.3 Hz, 3H,), ^13^C-NMR (101 MHz, CD_2_Cl_2_) δ, ppm: 174.2, 169.1, 151.2, 130.1, 126.7, 121.9, 81.9, 53.2, 35.7, 29.9, 26.1, 25.1, 16.0. HRMS (ESI-MS) m/z calculated for C_15_H_16_O_4_ [M+H]^+^ 261.1121, found 261.1125.

**Derivatization of P23**

**P23^deriv^:** Lactone **P23^deriv^** was isolated using **(*S,S*)-2^iPr^** in a 0.200 mmol scale. After completion of the catalysis, the reaction mixture was evaporated to complete dryness, dissolved in DCM (2 mL), and treated with phenol (37.6 mg, 3 equiv respect to the **S23**) and EDC·HCl (61 mg, 3 equiv respect to the **S23**). Τhe mixture was stirred overnight at room temperature. After concentration under reduced pressure, the crude mixture was directly loaded onto a chromatographic column and purified by flash chromatography on silica gel using hexane:EtOAc (25:1) to afford the esterified derivatized product **P23^deriv^** as a white solid (3 mg, 0.011 mmol, 6% yield over two steps). ^1^H-NMR (400 MHz, CD_2_Cl_2_) δ, ppm: 7.43 – 7.38 (m, 2H), 7.29 – 7.25 (m, 1H), 7.12 – 7.09 (m, 2H), 2.78 (dt, *J* = 11.5, 2.4 Hz, 1H), 2.48 – 2.42 (m, 1H), 2.02 – 1.98 (m, 2H), 2.02 – 1.98 (m, 1H) 1.53 (s, 3H), 1.59 – 1.46 (m, 1H), 1.27 – 1.26 (m, 3H), 1.07 (d, *J* = 6.3 Hz, 3H). ^13^C-NMR (101 MHz, CD_2_Cl_2_) δ, ppm: 173.9, 168.9, 151.2, 130.1, 126.7, 122.0, 84.9, 56.3, 47.0, 42.9, 37.1, 27.5, 25.2, 21.1. ${[\alpha]}_{D}^{20}$= +54.26 (c = 0.133, CHCl_3_). HRMS (ESI-MS) m/z calculated for C_16_H_18_O_4_ [M+H]^+^ 275.1278, found 275.1282.

**Derivatization of P24**

**P24^deriv^:** Lactone **P24** (28 mg, 0.15 mmol, 1 equiv.) dissolved in DCM (2 mL) and treated with phenol (20 mg, 1.5 equiv) and EDC·HCl (35 mg, 1.5 equiv). Τhe mixture was stirred overnight at room temperature. After concentration under reduced pressure, the crude mixture was directly loaded onto a chromatographic column and purified by flash chromatography on silica gel to afford the esterified product **P24^deriv^** as a white solid ^1^H-NMR (400 MHz, CDCl_3_) δ, ppm: 7.43 – 7.34 (m, 2H), 7.24 – 7.22 (m, 1H), 7.12 – 7.09 (m, 2H), 5.05 – 5.01 (m, 1H), 3.24 – 3.09 (m, 1H), 2.45 – 2.41 (m, 1H), 2.38 (d, *J* = 13.0 Hz, 1H), 2.21 – 2.09 (m, 2H), 1.81 – 1.69 (m, 5H). ^13^C-NMR (101 MHz, CDCl_3_) δ, ppm: 176.2, 169.5, 150.7, 129.6, 126.4, 121.4, 78.3, 54.6, 35.1, 34.0, 33.7, 23.9, 22.7. ${[\alpha]}_{D}^{20}$= +25.6 (c = 0.188, CHCl_3_). HRMS (ESI-MS) m/z calculated for C_15_H_16_O_4_ [M+Na]^+^ 283.0941, found 283.0943.

Cyclosyl-B column. GC temperature program: starting at 75°C for 0.5 minutes, then raised to 115°C at 10°C/min and hold 1 min. Then, raised to 130°C at 3°C/min and hold for 30 minutes. Then to 180°C at 3°C/min and hold 0 min. Then analysis time was 57.1 minutes. t_1_ = 18.0 min and t_2_ = 18.6 min.

Cyclosyl-B column. GC temperature program: starting at 75°C for 0.5 minutes, then raised to 115°C at 10°C/min and hold 1 min. Then, raised to 130°C at 3°C/min and hold for 30 minutes. Then to 180°C at 3°C/min and hold 0 min. Then analysis time was 57.1 minutes. t_1_ = 20.4 min and t_2_ = 20.9 min.

Cyclosyl-B column. GC temperature program: starting at 75°C for 0.5 minutes, then raised to 115°C at 10 °C/min and hold 1 min. Then, raised to 130°C at 3°C/min and hold for 30 minutes. Then to 180°C at 3°C/min and hold 0 min. Then analysis time was 57.1 minutes. t_1_ = 16.3 min and t_2_ = 16.8 min.

Cyclosyl-B column. GC temperature program: starting at 60°C for 30 minutes, then raised to 75°C at 0.5°C/min and hold 200 min. Then, raised to 80°C at 1 °C/min and hold for 200 minutes. Then to 90°C at 5°C/min and hold 20 min. Then to 220°C at 75°C/min and hold 0 min. Then analysis time was 488.7 minutes. (***minor diastereomer***) t_1_ = 323.3 min and t_2_ = 330.1 min. (***major diastereomer***) t_1_ = 386.1 min and t_2_ = 391.0min.

*Racemic*

*Racemic*

*Racemic*

*Racemic*

SFC analysis. Chiralpak IB, CO_2_:IPA 90:10, 35°C, flow rate = 1.2 mL/min, λ = 210 nm: The analysis time was 4 minutes. (***minor diastereomer***) t_1_ = 1.86 min and t_2_ = 2.36 min. (***major diastereomer***) t_1_ = 2.66 min and t_2_ = 3.00 min.

*Racemic*

*Racemic*

*Racemic*

*Racemic*

Cyclosyl-B column. GC temperature program: starting at 75°C for 0.5 minutes, then raised to 110°C at 2°C/min and hold 60 min. Then, raised to 12°C at 5°C/min and hold for 30 minutes. Then to 220°C at 5°C/min and hold 30 min. Then analysis time was 160.0 minutes. t_1_ = 79.7 min and t_2_ = 80.2 min.

SFC analysis. Chiralpak IG, CO_2_:IPA 90:10, 35°C, flow rate = 1.4 mL/min, λ = 220 nm: The analysis time was 4 minutes. (***major diastereomer***) t_1_ = 1.52 min and t_2_ = 1.77 min.

*Racemic*

*Racemic*

SFC analysis. Chiralpak IG, CO_2_:IPA 90:10, 35°C, flow rate = 1.2 mL/min, λ = 220 nm: The analysis time was 4 minutes. (***major diastereomer***) t_1_ = 1.38 min and t_2_ = 1.70 min.

*Racemic*

*Racemic*

SFC analysis. Chiralpak IJ, CO_2_:EtOH 85:15, 35°C, flow rate = 1.2 mL/min, λ = 210 nm: The analysis time was 4 minutes. (***minor diastereomer***) t_1_ = 2.01 min and t_2_ = 2.17 min. (***major diastereomer***) t_1_ = 2.46 min and t_2_ = 2.65 min.

*Racemic*

*Racemic*

*Racemic*

*Racemic*

SFC analysis. Chiralpak IA, CO_2_:MeOH 90:10, 35°C, flow rate = 2.0 mL/min, λ = 210 nm: The analysis time was 4 minutes. (***major diastereomer***) t_1_ = 2.16 min and t_2_ = 2.45 min.

*Racemic*

*Racemic*

SFC analysis. Chiralpak IJ, CO_2_:MeOH 97:3, 35°C, flow rate = 1.4 mL/min, λ = 210 nm: The analysis time was 4 minutes. (***minor diastereomer***) t_1_ = 2.63 min and t_2_ = 2.92 min.

*Racemic*

*Racemic*

SFC analysis. Chiralpak IJ, CO_2_:MeOH 95:5, 35°C, flow rate = 1.4 mL/min, λ = 210 nm: The analysis time was 4 minutes. (***major diastereomer***) t_1_ = 2.44 min and t_2_ = 2.74 min.

*Racemic*

*Racemic*

SFC analysis. Chiralpak IG, CO_2_:IPA 90:10, 35°C, flow rate = 1.5 mL/min, λ = 210 nm: The analysis time was 4 minutes. (***minor diastereomer***) t_1_ = 1.57 min and t_2_ = 2.79 min.

*Racemic*

*Racemic*

SFC analysis. Chiralpak IG, CO_2_:IPA 80:20, 35°C, flow rate = 1.2 mL/min, λ = 210 nm: The analysis time was 4 minutes. (***major diastereomer***) t_1_ = 1.19 min and t_2_ = 1.76 min.

*Racemic*

*Racemic*

Cyclosyl-B column. GC temperature program: starting at 75°C for 0.5 minutes, then raised to 115°C at 10 °C/min and hold 1 min. Then, raised to 130°C at 3°C/min and hold for 30 minutes. Then to 180°C at 3°C/min and hold 0 min. Then analysis time was 57.1 minutes. t_1_ = 25.2 min and t_2_ = 26.4 min.

Cyclosyl-B column. GC temperature program: starting at 50°C for 0.5 minutes, then raised to 85°C at 0.5°C/min and hold 150 min. Then, raised to 90°C at 1°C/min and hold for 200 minutes. Then to 220°C at 5°C/min and hold 2 min. Then analysis time was 453.5 minutes. t_1_ = 410.6 min and t_2_ = 416.17 min.

SFC analysis. Chiralpak IG, CO_2_:EtOH 80:20, 35°C, flow rate = 1.2 mL/min, λ = 220 nm: The analysis time was 4 minutes. t_1_ = 2.51 min and t_2_ = 3.02 min.

*Racemic*

*Racemic*

SFC analysis. Chiralpak IG, CO_2_:IPA 90:10, 35°C, flow rate = 1.5 mL/min, λ = 220 nm: The analysis time was 6 minutes. t_1_ = 4.23 min and t_2_ = 5.28 min.

*Racemic*

*Racemic*

Cyclosyl-B column. GC temperature program: starting at 75°C for 0.5 minutes, then raised to 120°C at 10°C/min and hold 60 min. Then, raised to 220°C at 5°C/min and hold for 2 minutes. Then analysis time was 87.0 minutes. t_1_ = 79.7 min and t_2_ = 79.8 min.

Cyclosyl-B column. GC temperature program: starting at 75°C for 0.5 minutes, then raised to 115°C at 10°C/min and hold 1 min. Then, raised to 130°C at 3°C/min and hold for 30 minutes. Then to 180°C at 3°C/min and hold 0 min. Then analysis time was 57.1 minutes. t_1_ = 9.79 min and t_2_ = 10.0 min.

SFC analysis. Chiralpak IC, CO_2_:MeOH 85:15, 35°C, flow rate = 1.2 mL/min, λ = 210 nm: The analysis time was 4 minutes. t_1_ = 1.17 min and t_2_ = 1.57 min.

*Racemic*

*Racemic*

SFC analysis. Chiralpak IC, CO_2_:MeOH 85:15, 35°C, flow rate = 1.2 mL/min, λ = 210 nm: The analysis time was 4 minutes. t_1_ = 1.28 min and t_2_ = 1.53 min.

*Racemic*

*Racemic*

SFC analysis. Chiralpak IB, CO_2_:IPA 90:10, 35°C, flow rate = 1.2 mL/min, λ = 210 nm: The analysis time was 4 minutes. (***minor diastereomer***) t_1_ = 1.86 min and t_2_ = 2.36 min. (***major diastereomer***) t_1_ = 2.66 min and t_2_ = 3.00 min.

*Racemic*

*Racemic*

*Racemic*

*Racemic*

SFC analysis. Chiralpak IC, CO_2_:IPA 82:18, 35°C, flow rate = 1.0 mL/min, λ = 210 nm: The analysis time was 6 minutes. t_1_ = 1.21 min and t_2_ = 1.77 min.

*Racemic*

*Racemic*

SFC analysis. Chiralpak IC, CO_2_:EtOH 90:10, 35°C, flow rate = 1.2 mL/min, λ = 214 nm: The analysis time was 4 minutes. t_1_ = 1.21 min and t_2_ = 1.77 min.

*Racemic*

*Racemic*

Cyclosyl-B column. GC temperature program: starting at 50°C for 0.5 minutes, then raised to 85°C at 0.5°C/min and hold 150 min. Then, raised to 90°C at 1°C/min and hold for 200 minutes. Then to 220°C at 5°C/min and hold 2 min. Then analysis time was 453.5 minutes. t_1_ = 431.8 min and t_2_ = 432.5 min.

Cyclosyl-B column. GC temperature program: starting at 50°C for 0.5 minutes, then raised to 85°C at 0.5°C/min and hold 150 min. Then, raised to 90°C at 1°C/min and hold for 200 minutes. Then to 220°C at 5°C/min and hold 2 min. Then analysis time was 453.5 minutes. t_1_ = 448.9 min and t_2_ = 449.1 min.

SFC analysis. Chiralpak IB, CO_2_:MeOH 97:3, 35°C, flow rate = 1.0 mL/min, λ = 210 nm: The analysis time was 5 minutes. t_1_ = 3.47 min and t_2_ = 3.79 min.

*Racemic*

*Racemic*

SFC analysis. Chiralpak IC, CO_2_:IPA 92:8, 10°C, flow rate = 1.2 mL/min, λ = 210 nm: The analysis time was 5 minutes. t_1_ = 2.86 min and t_2_ = 3.31 min.

*Racemic*

*Racemic*

SFC analysis. Chiralpak IG, CO_2_:EtOH 85:15, 35°C, flow rate = 1.2 mL/min, λ = 210 nm: The analysis time was 5 minutes. t_1_ = 2.26 min and t_2_ = 2.45 min.

*Racemic*

*Racemic*

Cyclosyl-B column. GC temperature program: starting at 75°C for 0.5 minutes, then raised to 110°C at 5°C/min and hold 60 min. Then, raised to 240°C at 20°C/min and hold for 0 minutes. Then to 180°C at 3°C/min and hold 0 min. Then analysis time was 74.0 minutes. t_1_ = 39.6 min and t_2_ = 40.5 min.

SFC analysis. Chiralpak IC, CO_2_:MeOH 85:15, 35°C, flow rate = 1.2 mL/min, λ = 240 nm: The analysis time was 5 minutes. t_1_ = 2.69 min and t_2_ = 3.55 min.

*Racemic*

*Racemic*

Cyclosyl-B column. GC temperature program: starting at 75°C for 0.5 minutes, then raised to 120°C at 10°C/min and hold 60 min. Then, raised to 220°C at 5°C/min and hold for 30 minutes. Then to 180°C at 3°C/min and hold 0 min. Then analysis time was 115.0 minutes. t_1_ = 73.6 min and t_2_ = 74.3 min.

**16. References**

[1] R. V. Ottenbacher, K. P. Bryliakov, E. P. Talsi, *Adv. Synth. Catal* **2011**, *353*, 885-889.

[2] M. Milan, M. Bietti, M. Costas, *ACS Cent. Sci.* **2017**, *3*, 196-204.

[3] W. Sun, Q. Sun, *Acc. Chem. Res.* **2019**, *52*, 2370-2381.

[4] A. Call, G. Capocasa, A. Palone, L. Vicens, E. Aparicio, N. Choukairi Afailal, N. Siakavaras, M. E. López Saló, M. Bietti, M. Costas, *J. Am. Chem. Soc.* **2023**, *145*, 18094-18103.

[5] F. Malmedy, T. Wirth, *Eur. J. Org. Chem.* **2017**, *2017*, 786-789.

[6] K. D. Collins, J. M. Oliveira, G. Guazzelli, B. Sautier, S. De Grazia, H. Matsubara, M. Helliwell, D. J. Procter, *Chem. Eur. J.* **2010**, *16*, 10240-10249.

[7] K.-N. Lau, H.-F. Chow, M.-C. Chan, K.-W. Wong, *Angew. Chem. Int. Ed.* **2008**, *47*, 6912-6916.

[8] I. Tellitu, I. Beitia, M. Díaz, A. Alonso, I. Moreno, E. Domínguez, *Tetrahedron* **2015**, *71*, 8251-8255.

[9] M. D. Mihovilovic, T. C. M. Fischer, P. Stanetty, *Molecules* **2006**, *11*, 357-364.

[10] M. Mato, C. García-Morales, A. M. Echavarren, *ACS Catal.* **2020**, *10*, 3564-3570.

[11] B. Yadav, B. Baire, *Adv. Synth.Catal.* **2022**, *364*, 4305-4309.

[12] X. Cong, Q. Zhuo, N. Hao, Z. Mo, G. Zhan, M. Nishiura, Z. Hou, *Angew. Chem. Int. Ed.* **2022**, *61*, e202115996.

[13] R. Sang, P. Kucmierczyk, R. Dühren, R. Razzaq, K. Dong, J. Liu, R. Franke, R. Jackstell, M. Beller, *Angew. Chem. Int. Ed.* **2019**, *58*, 14365-14373.

[14] D. J. R. Velaga, R. K. Arigela, S. Kokatnur, S. Jawlekar, R. R. Budhdev, N. L. R. Susarla, S. Ramakrishnan, A. Manda, S. K. Komati, G. C. Senadi, A. Maruthapillai, R. Bandichhor, *Org. Process Res. Dev.* **2025**, *29*, 2559-2568.

[15] K. S. McClymont, F.-Y. Wang, A. Minakar, P. S. Baran, *J. Am. Chem. Soc.* **2020**, *142*, 8608-8613.

[16] C. Xie, D. Han, Y. Hu, J. Liu, T. Xie, *Tetrahedron Lett.* **2010**, *51*, 5238-5241.

[17] S. Kotha, M. K. Dipak, S. M. Mobin, *Tetrahedron* **2011**, *67*, 4616-4619.

[18] C.-G. Yang, N. W. Reich, Z. Shi, C. He, *Org. Lett.* **2005**, *7*, 4553-4556.

[19] Z. Qureshi, H. Weinstabl, M. Suhartono, H. Liu, P. Thesmar, M. Lautens, *Eur. J. Org. Chem.* **2014**, *2014*, 4053-4069.

[20] Y. Kobayashi, M. Nuruzzaman, N. Ogawa, N. Maeda, K. Miyoshi, H. Nonaka, T. Murakami, *J. Org. Chem.* **2025**, *90*, 3034-3042.

[21] R. I. Vasiuta, M. V. Gorichko, *Tetrahedron Lett.* **2014**, *55*, 466-468.

[22] J. Wilent, K. S. Petersen, *J. Org. Chem.* **2014**, *79*, 2303-2307.

[23] C. Berini, O. Navarro, *Chem. Comm.* **2012**, *48*, 1538-1540.
